# Hexagonal boron nitride: a review of the emerging material platform for single-photon sources and the spin–photon interface

**DOI:** 10.3762/bjnano.11.61

**Published:** 2020-05-08

**Authors:** Stefania Castelletto, Faraz A Inam, Shin-ichiro Sato, Alberto Boretti

**Affiliations:** 1 School of Engineering, RMIT University, Melbourne, Victoria 3000, Australia; 2 Dept. of Physics, Aligarh Muslim University, Aligarh, U.P. 202002, India; 3 National Institutes for Quantum and Radiological Science and Technology, Takasaki, Gunma, 370-1292, Japan; 4 Mechanical Engineering Department, College of Engineering, Prince Mohammad Bin Fahd University, Al Khobar 31952, Kingdom of Saudi Arabia

**Keywords:** boron nitride, color centers, quantum applications, quantum properties

## Abstract

Single-photon sources and their optical spin readout are at the core of applications in quantum communication, quantum computation, and quantum sensing. Their integration in photonic structures such as photonic crystals, microdisks, microring resonators, and nanopillars is essential for their deployment in quantum technologies. While there are currently only two material platforms (diamond and silicon carbide) with proven single-photon emission from the visible to infrared, a quantum spin–photon interface, and ancilla qubits, it is expected that other material platforms could emerge with similar characteristics in the near future. These two materials also naturally lead to monolithic integrated photonics as both are good photonic materials. While so far the verification of single-photon sources was based on discovery, assignment and then assessment and control of their quantum properties for applications, a better approach could be to identify applications and then search for the material that could address the requirements of the application in terms of quantum properties of the defects. This approach is quite difficult as it is based mostly on the reliability of modeling and predicting of color center properties in various materials, and their experimental verification is challenging. In this paper, we review some recent advances in an emerging material, low-dimensional (2D, 1D, 0D) hexagonal boron nitride (h-BN), which could lead to establishing such a platform. We highlight the recent achievements of the specific material for the expected applications in quantum technologies, indicating complementary outstanding properties compared to the other 3D bulk materials.

## Review

### Introduction

Point defects (impurity atoms or complex of atoms) in solids are recognized elementary units for various quantum technology applications, such as quantum information science [1], quantum sensing [2], quantum cryptography [3], and quantum computing [4–5]. After the discovery and assessment of their quantum properties, some of these defects became prominent examples of material platforms for quantum photonics [6–10] and spin–photon interfaces for remote spin–photon entanglement with available nuclear spins as ancilla qubits for quantum memory [11–12]. These include the nitrogen-vacancy (NV) center in diamond [13], the silicon-vacancy center in diamond [14–16], the germanium-vacancy center in diamond [17], the divacancy (DV) in silicon carbide (SiC) [18–20], the silicon monovacancy in SiC [21–23], the carbon antisite vacancy pair in SiC [24–25], the silicon vacancy and nitrogen (N) atom on an adjacent carbon site in SiC [26–28], and rare-earth impurities in complex oxides [29]. While the NV center in diamond is currently the preferred platform for implementing quantum sensing and quantum computing approaches, the recent emergence of other interesting color centers in diamond itself [16,30–31] and in other materials indicates that indeed NV is not optimal for many applications, neither it is unique. The search for other platforms is mainly motivated by either scalability or manufacturability within a monolithic platform fabrication or opportunities to facilitate hybrid integration of quantum materials with existing mature devices (hybrid approach).

However, there are other materials that are at an even more emerging stage of development that can serve as alternative material platforms. These are generally the wide-bandgap group II–VI and III–V materials, such GaN [32–34] and ZnO [35–37], and low-dimensional van der Waals materials, including the transition metal dichalcogenide (TMD) materials, such as WSe_2_ and WS_2_ [38–41], hexagonal boron nitride (h-BN) [42–47] and WO_3_ [48].

A summary of quantum point defects identified in various emerging materials is provided in Table 1 for a quick comparison. Details of h-BN are then discussed in the specific sections of this paper, while we remind the readers of recent reviews on the other materials or emerging point defects in diamond [10,31,42,59–61] and SiC [7,11,62].

**Table 1:** Summary of recent examples of single-photon emitters (SPEs) in various emerging materials. RT = room temperature.

Platform	Bandgap (eV)	Photo- luminescence (nm)	Optical excitation (nm)	*T* (K)	Brightness (kcts/s)	Lifetime (ns)	Assignment

h-BN (single and multilayers, single crystal)	6	569–750	532, 675 or CL	RT	20–7000	1–3	various from different point defects and stacking faults
WO_3_ multilayers [48]	3.6	620–730	532	RT	350	3.5–4.4	deep charge state
WS_2_ (single and multilayers) [49]	2	610–680	514.5	10	–	–	–
GaSe(multilayers) [50]	2.1	660	–	10	<0.1	5–22	exciton/biexciton
MoSe_2_ (flakes) [51]	0.85–1.5 (direct)	765–772	675	4	0.6 [39]	1 [39]	quantum dot-like emission
WSe_2_ (monolayer) [38,40,52–54]	1.7	700–800	532	4	6–37 [40,55]	0.6–2.5, 4.14 [55]	quantum dot-like emission
GaN (epilayers) [33]	3.4	600–750	532	RT	500	4.7	cubic inclusions in hexagonal
GaN epilayers [56]	3.4	1085–1340	950	RT	690	0.74	point defect optically active in the proximity of cubic inclusions in the hexagonal lattice
ZnO (thin films and nanoparticles) [35,37]	3.3	660–793	532	RT	184	4.16	tentatively to *V*_O_ or *V*_Zn_, most common defects in ZnO
TiO_2_ (thin film and nanoparticles) [57]	3.05	600–700	532	RT	<60	≈0.5 (large non-radiative decay)	unknown
ZnS (nanoparticles) [58]	3.6–3.9	≈640	532	RT	140	2.2	unknown

In this review, the material of focus is h-BN. The current progress indicates h-BN is distinguishing itself with great potential as a quantum point defect material as well as a photonics material [63]. This choice of material is supported on one hand by the existence of room-temperature single-photon (SP) emission, with the highest brightness and on the other hand by the ability of coherent spin control of some point defects at room temperature. Other materials currently may not exhibit room-temperature SP emission or there is no information about their potential paramagnetic defects that can be optically manipulated. MoS_2_ and h-BN are the most studied 2D platforms. MoS_2_ and h-BN have several nuclear spin atoms with large nuclear spins and concentrations; however, suitable impurities have not yet been recognized. As such, ancilla qubits such as nuclear spins that can couple with the point defects for quantum memories in a future quantum network appear to be still an element under investigation among these emerging materials even if hyperfine interaction with nearby nuclear spins has been observed.

To investigate materials in terms of tailoring their quantum point defects, the historical approach consists in presenting previous studies of photoluminescent point defects, their ensemble photoluminescence (PL) characterization and generation using ions, electrons or other irradiation methods, the observation of ensemble paramagnetic properties and their optically detected magnetic resonance by applying a high magnetic field until the isolation of single defects and eventually their optical spin coherent control. Weber et al. [5] took the first step in this direction, showing how basic considerations of host properties (e.g., nuclear spin isotopes, bandgap, and spin–orbit coupling) can guide the identification of quantum point defects analogous to the diamond NV center, elevating SiC as such a host, with now many SP and spin-controlled point defects in this material [62].

The host material synthesis is also relevant, as the possibility to study single NVs in diamond and similar defects in SiC is based on the commercial availability of high-purity materials. Scalability is instead related to the host material wafer-scale fabrication. This must be available at the same level of purity, with doping control and electronic compatibility, as well as scalable methodologies to create a large number of arrays of point defects with controlled emission to remotely entangle them and couple to ancilla qubits for quantum memory to build an example future quantum network [64]. This should preferably be in a monolithic structure to reduce manufacturability limitations and materials mismatch.

In an ultrathin 2D material like h-BN, however, conventional characterization is difficult due to the sensitivity limited to large volume sampling, while isolation of single defects or their ab-initio modeling can be a better guide. This holds even if the assignment to specific defects may be challenging without being able to address their ensemble generation. For these materials, atomic resolution methods could be used, such as atom probe tomography, providing sub-nanometer spatial information of the chemical composition, scanning tunneling electron microscope (STEM) imaging and spectroscopy at low beam energy [65], enabling the characterization of individual defects in h-BN, and atomic electron tomography. However, all these methods also have limitations such as sample damaging or even destruction. Further 2D materials are more likely to be manufactured within existing devices as such following a hybrid approach. Hybrid approaches are the ones mostly pursued; however, they suffer from poor performance due to mismatch/compatibility of the active quantum source and the device in use, and they have limitations in terms of scalability.

The type of point defects that should be addressed is also a key element and is generally substitutional dopants, native vacancies, and dopant-vacancy complexes. The space of possible defects is however quite large especially in compound materials where the antisite defects are also quite common.

In the following sections, we will follow the above-described approaches to review the current performance of quantum point defects in h-BN, identifying the pathways for their applications both in hybrid approaches for 2D, 1D, and 0D nanomaterials or a monolithic approach for the bulk material.

### Single-photon sources and their assessment criteria

A single-photon source (SPS) is an optical device deterministically emitting photons. An SPS should have sub-Poisonnian statistics for photon emission, i.e., emitting an anti-bunched stream of single photons well separated in time. The photon statistics of an SPS can be simply understood in terms of the number (or Fock) state description of light. For light in a mode of the number state, the second order correlation function is given by [66]

[1]
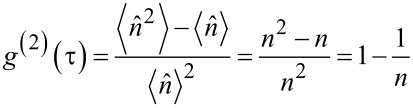


for all states except the vacuum state. For light in the SP state (*n* = 1), the *g*^(2)^(τ) value will be zero. Such light can be understood as a stationary SP in a single optical mode while a realistic SPS is expected to emit photons at separate time intervals given by the excitation source rate with no coincidence between the different photon bursts. The above analysis applies for τ = 0. With the increase in time (τ), the possibility of subsequent photon emission events will increase, leading to an increase in the *g*^(2)^(τ) value.

A practical SPS can be realized by isolating a single quantum emitter in the form of a single atom, a single molecule, a single solid-state color center or a single quantum dot. The spontaneous emission from these isolated systems has the characteristics of either a 2-level [67] or a 3-level atomic model [68].

**Figure 1:**
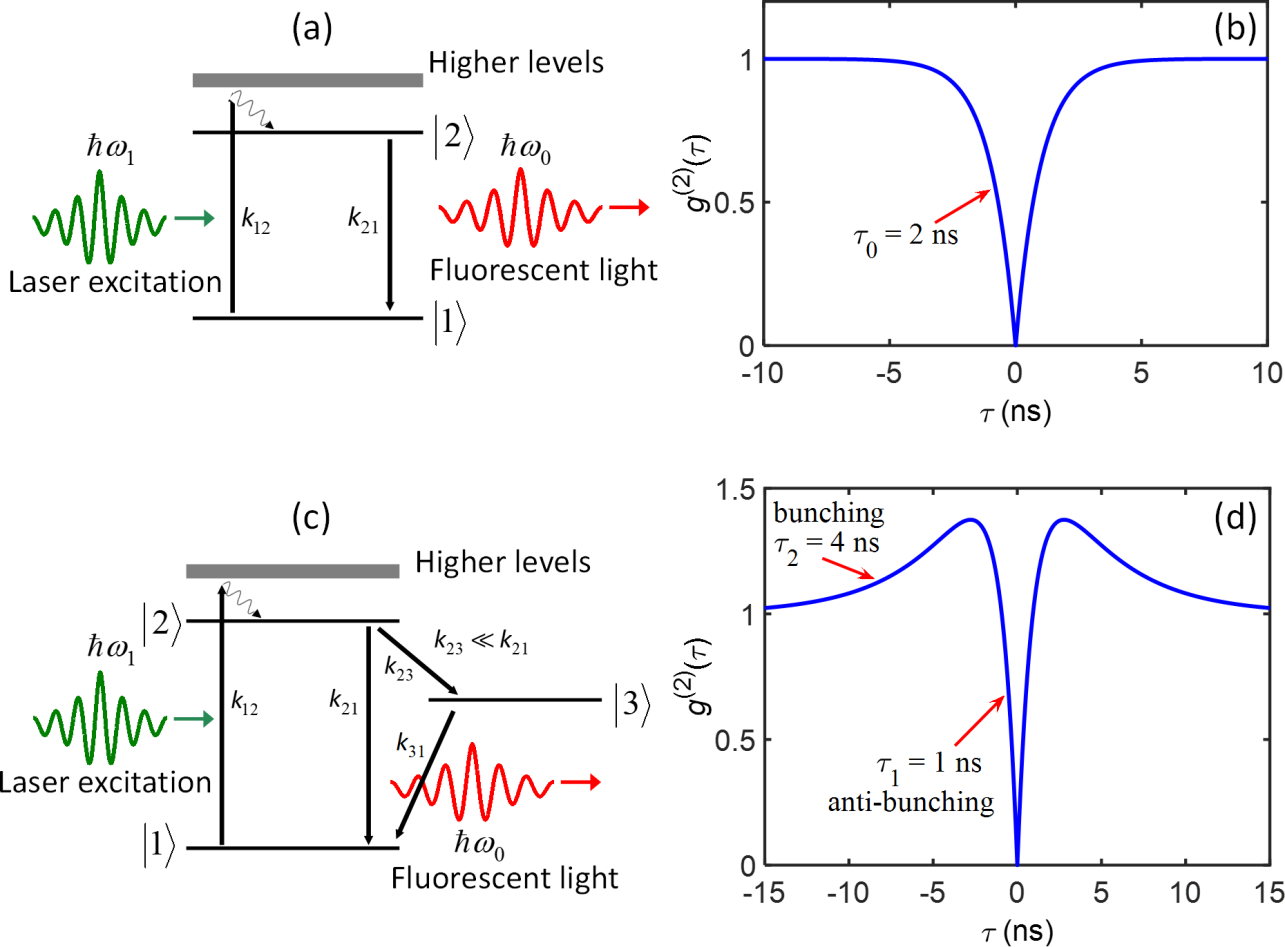
(a) Schematic representation of spontaneous emission from a non-resonantly driven 2-level system. (b) The second order correlation function for a 2-level system, *g*^(2)^(τ). (c) Schematic representation of spontaneous emission from a non-resonantly driven 3-level system. (d) The second order correlation function for a 3-level system.

A 2-level system representation is shown in Figure 1a. It comprises a ground state |1⟩ and an excited state |2⟩ with an energy difference of ℏω_0_. From the ground state |1⟩, the system is driven to the higher levels by a laser source with frequency ω_1_ > ω_0_. The pump rate coefficient *k*_12_ is proportional to the excitation laser power.

The system then instantly relaxes to the excited state |2⟩. The system then decays to the ground state |1⟩ via spontaneous emission, emitting a photon of energy ℏω_0_. The coefficient *k*_21_ gives the decay rate, and the excited state lifetime, τ_0_, is the reciprocal of the decay rate, τ_0_ = 1/*k*_21_. If all transitions described by *k*_21_ are radiative, the decay rate will be the same as the spontaneous emission rate (SER). As described in [69], neglecting all the atomic coherences, the rate equations for the system are given by:

[2]
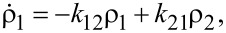


[3]
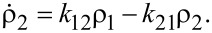


Here ρ_1,2_ represents the population of states |1⟩ and |2⟩ and should satisfy the completeness condition for the system to be in either of the two states ρ_1_ + ρ_2_ = 1. The solution of the above rate equations gives,

[4]
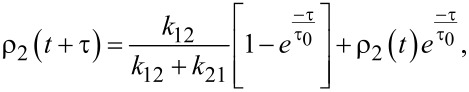


with

[5]
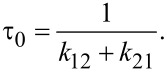


In the equilibrium condition, 
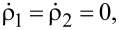
 the steady-state population of the state |2⟩ is given by

[6]
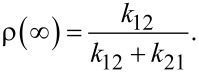


As the system is in the ground state initially,

[7]
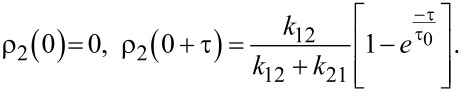


To correlate the transition rates with the photon detection events, let us now introduce the atomic level transition operators, 
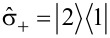
 and 
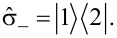
 The relationship between the annihilation operator for the light field and the transition operator is expressed as [66]:

[8]
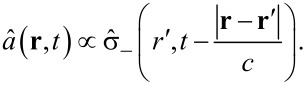


This implies that the detection event of a photon by a detector placed at a position **r** at time *t* is coupled to an atomic transition of an emitter placed at a position **r**’ from state |2⟩ to |1⟩ at time *t* − |**r** − **r**’|/*c*. Since the expectation value of the photon number operator 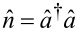 corresponds to the number of photons in the system, similarly, the expectation value of the operator 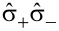 should correspond to the population of the state |2⟩.

Making use of the above discussion, the second order correlation function for the fluorescence light of a 2-level system can be written as:

[9]
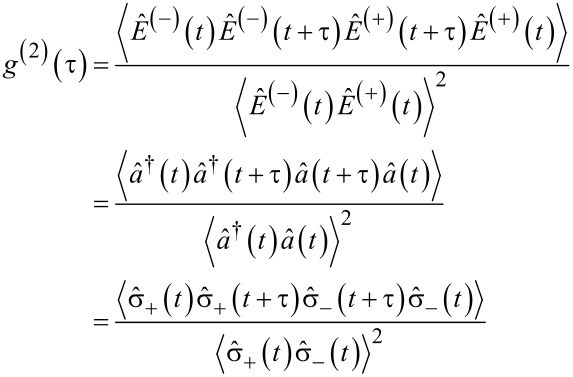


Using the quantum regression theorem [70], we get:

[10] $$\begin{array}{l} \langle {\hat{\sigma }}_{+}\left(t\right){\hat{\sigma }}_{+}\left(t+\tau \right){\hat{\sigma }}_{-}\left(t+\tau \right){\hat{\sigma }}_{-}\left(t\right)\rangle \\ \;\;\;=\sum_{i}\alpha_{i}\left(\tau \right)\langle {\hat{\sigma }}_{+}\left(t\right){\hat{A}}_{i}\left(t\right){\hat{\sigma }}_{-}\left(t\right)\rangle , \end{array}$$

where

[11]
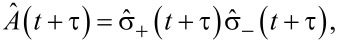


with the evolution of 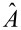 written as

[12]
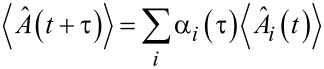


for some operator 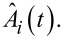 As the expectation value 
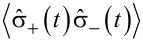
 gives the population of the state |2⟩, i.e., ρ_2_(*t*), we have

[13] $$\begin{array}{l} \langle {\hat{\sigma }}_{+}\left(t+\tau \right){\hat{\sigma }}_{-}\left(t+\tau \right)\rangle \\ \;\;\;\;=\frac{k_{12}}{k_{12}+k_{21}}\left[1-e^{\frac{-\tau }{\tau_{0}}}\right]+e^{\frac{-\tau }{\tau_{0}}}\langle {\hat{\sigma }}_{+}\left(t\right){\hat{\sigma }}_{-}\left(t\right)\rangle . \end{array}$$

On comparing the above equation with the evolution expression for 
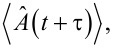
 one can identify

[14]
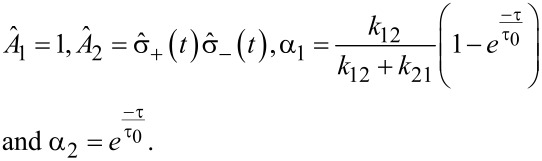


By using these identities, we obtain:

[15]
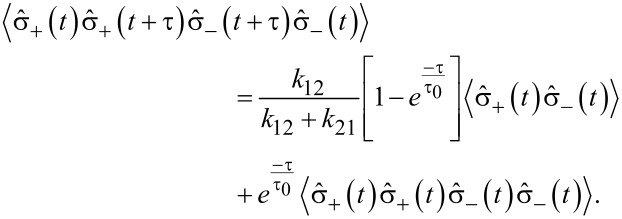


As 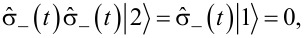 with |1⟩ being the ground state, then

[16]
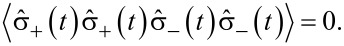


Considering 
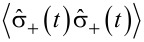
 as the population of the state |2⟩ in equilibrium, i.e., ρ_2_(∞), the second order correlation function for the fluorescence light of a 2-level system is

[17]
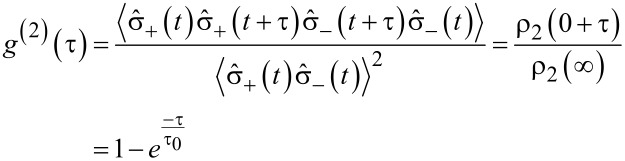


at τ = 0, *g*^(2)^(τ) = 0. Therefore, a non-resonantly driven 2-level system behaves as an SPS, emitting light in the SP state. The anti-bunching decay time τ_0_ for zero excitation power (i.e., *k*_12_ = 0) is τ_0_(*P* → 0) = 1/*k*_12_. Since *k*_12_ is the decay time of the excited state, τ_0_(*P* → 0) is the fluorescence lifetime of the 2-level emitter.

A 3-level system comprises an intermediate meta-stable state together with the ground and the excited states. Figure 1c shows the representation of a 3-level system. The state |3⟩ is a metastable state, i.e., transitions between it and the ground state |1⟩ will occur at a much longer time scale compared to transitions between the excited state |2⟩ and the ground state |1⟩. In the physical system, including the diamond NV centers, the ground state |1⟩, and the excited state |2⟩ are spin-triplet states and the meta-stable state is a spin-singlet state. The system non-radiatively relaxes between the excited triplet state |2⟩ and the singlet meta-stable state |3⟩. These non-radiative relaxation processes between the different spin states are known as inter-system crossing (ISC). The decay process between the different spin states occurs at a much slower rate and is mediated by the spin–orbit coupling. Therefore, *k*_23_ ≪ *k*_21_. This results in the metastable nature of the state |3⟩. From the metastable state |3⟩, the system finally relaxes to the ground state via phosphorescence. Fluorescence occurs when the system relaxes from state *v*|2⟩ to state *v*|1⟩ via spontaneous emission, emitting a photon of energy ℏω_0_. In the same approximation as the 2-level systems, the rate equations for the 3-level system are:

[18]
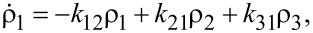


[19]
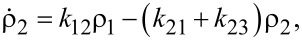


[20]
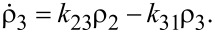


The second order autocorrelation function for the three-level system is given by [67,71]:

[21]
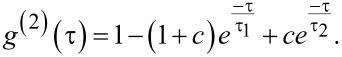


Here the decay times τ_1_, τ_2_ and the coefficient *c* are given by

[22]
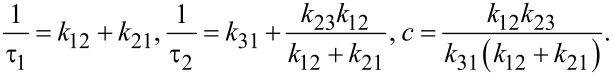


In the above *g*^(2)^(τ) function, τ_1_ represents the anti-bunching decay time and τ_1_ represents the bunching process (Figure 1d). This is because the rates *k*_23_ and *k*_31_ are much smaller compared to *k*_21_, τ_1_ ≪ τ_2_. This means that the antibunching process occurs at a much faster time scale compared to the bunching process.

### Single-photon source measurements

Single-photon emission (SPE) is assessed by the indirect measurement of the photon correlation function, which is extracted from the histogram of the time arrivals of consecutive photons at the input of a 50:50 beam splitter. This is based on the Hanbury Brown and Twiss (HBT) interferometer [72]. The HBT interferometer measures the correlation in the intensity fluctuations of the light incident on the 50:50 beam splitter. The incoming light is equally split along the transmitted and the reflected paths onto the detectors (avalanche photodiodes (APD)) placed along both the paths. The electrical output of the photodiodes is then analysed by a time-correlator with a time delay introduced in one of the paths. The time correlator (a time-correlated SP counting module) measures the delay between the subsequent photon detection events by the two detectors in the histogram mode. The photon detection event by the non-time-delayed detector acts as a start signal with the subsequent photon detection event by the time-delayed detector acting as a stop signal. Coincidence counts for the two-photon detection events are plotted as a function of time with the time-interval histograms building up in real-time. These coincidences are collected over enough time duration to extract a decent coincidence curve for the incident light with acceptable noise limits. The total collection time is dependent on the intensity of the incoming light, being very large for dim or low-intensity light and very small for high-intensity light.

This raw coincidence curve for the detection events on the two detectors also contains events from the background emission. The background noise comes from the dark counts on the two detectors as well as from nearby emitters which are not resolved by the optical imaging system. A small fraction of the background counts may also come from the laser back reflection that passes through the filter. The coincidence data is background corrected and then normalized to obtain the intensity correlation function [73]. For this, the raw correlation events *c*(*t*) collected over a time duration *T* and within time bins of width *w* are normalized to that of a Poissonian source: *C_N_*(τ) = *c*(τ)/(*N*_1_*N*_2_*wT*), here *N*_1,2_ are count rates of each detector. The product *N*_1_*N*_2_*T* is the total number of coincidence counts collected over a time *T*. This product is multiplied with time bin width *w* to obtain the normalization factor *N*_1_*N*_2_*wT*. The normalized correlation data is then corrected for the background signal to yield the intensity or second order correlation function:

[23]
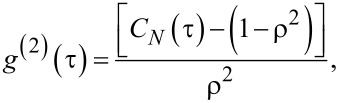


where

[24]
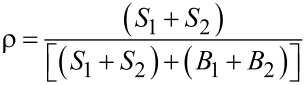


is the signal to background ratio with *S*_1,2_ and *B*_1,2_ being signal and background counts on each detector (*S*_1,2_ = *N*_1,2_ – *B*_1,2_).

Experimentally at delay time τ = 0, the ideal SPE *g*^(2)^(0) = 1 – 1/*N*, where *N* is the number of emitters. SPE is characterized by an ideal *g*^(2)^(0) = 0, while practically it is assumed to be single emitter if the *g*^(2)^(0) = < 0.5, two emitters when *g*^(2)^(0) ≈ 0.5, 3 emitters *g*^(2)^(0) ≈ 0.67, and so on. In fact, due to the photon counts background and finite response time of the correlation measurements are limited today by the convolution of the time response of the two SP detectors, *g*^(2)^(0) ≠ 0, in the case of the SP emitter. The purity of the SPE will be given by the value of *g*^(2)^(0).

For practical applications, it is required that the emission from an SPS should be very bright and highly directional and should lie within the collection solid angle of the detection system. A bright SPS should have a very fast radiative decay process resulting in very short lifetimes. A large enhancement in spontaneous emission rate (SER) is achieved by coupling the emitter’s emission to the resonance modes of photonics/plasmonic structures. These resonator structures provide the emitter with an enhanced local density of optical states (LDOS) for the emission to couple to, thus enhancing the radiative decay process [74]. The enhancement in the SER is quantified in terms of the Purcell factor, which is defined as the ratio of the total SER of the emitter in the resonator to its SER in a vacuum [74]. These photonic/plasmonic structures can also act as antennas, effectively out-coupling the emitter’s radiation into the far-field as well as providing directionality to the emitter’s far-field radiation pattern. The effectiveness of any resonator/antenna scheme is quantified in terms of both the Purcell enhancement and the collection efficiency (CE). The CE is defined as the ratio of the total power incident within the solid angle of the collection objective lens to the total power radiated by the emitter into the far-field, i.e., the power collected by the collection system to the total power radiated by the emitter into the far-field [75–77]. As the total power radiated into the far-field is dependent on the quantum efficiency (QE) of emission, which is the ratio of the radiative decay rate to the total decay rate and includes all the pathways for non-radiative decays [78–79], the CE is the product of the QE and the fraction of the far-field radiated power directed into the numerical aperture (NA) of the collection objective lens. A high, near unity CE value will ensure that the decay process is largely radiative with the emission being highly directional. Enhancement in both the Purcell factor and the CE will together significantly enhance the photon collection/extraction rate from the emitter.

### Single-photon source assessment criteria

An ideal SPS should emit per excitation pulse only one photon, with an ideally GHz range repetition rate, to permit fast and secure communication. There are several key criteria to benchmark the performance of a solid-state SPS to be considered suitable for applications in quantum technology [7–8,80]. These include the following: photo-stable emission without blinking or photo-bleaching; narrow bandwidth with most of the emission in the zero phonon line (ZPL), which is spectrally stable with no broadening and limited spectral diffusion, delivering a Fourier transform (FT) limited emission (spectral broadening equal to the inverse of the radiative optical transition lifetime) and thus photon indistinguishability; high purity, i.e., with *g*^(2)^(0) close to 0; high brightness (ideally on average 1 photon/pulse); and short lifetime and thus high repetition rate (>GHz), room temperature operation, high extraction efficiency (>90%). The SPS spectral purity can be measured with high-resolution photoluminescence (PL) excitation, (PLE). Photon indistinguishability is assessed when two photons from this source are identical for all photon degrees of freedom, namely spectral, spatial and polarization. It has been demonstrated by Hong–Ou–Mandel (HOM) interference (see [81]) that when two identical SP pulses from the same source or two different sources are sent to the two 50:50 beam splitter inputs, they will exit the beam splitter on the same output port. If two detectors are placed at the output, only one detector will register the signal, producing a dip in the coincidence counts. When the identical input SPs perfectly overlap in time on this beam splitter, the coincidence counts of the detectors will tend to zero, producing a coincidence dip directly related to the spectral broadening of the single-photon wave packet. As such the measure of photon indistinguishability is given by the mean SP pulse wave-packet overlap parameter *M*, with *M* = 1 indicating perfect indistinguishability. Photon indistinguishability is relevant for optical quantum technologies to implement two-photon quantum gates or to engineer entangled gates. Quantum entanglement is essential in quantum algorithms or quantum repeaters for long-distance quantum communication. Two-photon entanglement can be engineered using the quantum interference of two SP wave-packets. Similarly, for solid-state quantum computation architecture and related quantum networks [64], where quantum gates are achieved via electron spins while quantum memory is based on ancillary nuclear spins, spin–photon entanglement distribution is achieved based on SP indistinguishability and quantum interferometry.

SPS brightness is defined as the probability to have an SP per pulse. High SPS brightness combined with high source repetition rate, high transmission of the optical network and high detector efficiencies, contribute to the final speed of quantum communication or computation protocols.

For their integration into devices, the desirable criteria of SPSs are their potential to be electrically driven and their scalability in terms of their fabrication and integration at a high density on a wafer size substrate.

Other desirable criteria for spin–photon-phonon entanglement distribution are high electron spin coherence time *T*_2_ (approaching *T*_2_ spin-lattice relaxation time) and strain and electrical control of the spin transition and optical transition resonances. The key requirements can be restrictive depending on the applications as outlined in Table 2. In Table 2 the SPS criteria are described for specific application requirements.

**Table 2:** Characteristics of SPEs and spin–photon interface properties needed for specific applications. Purity, *g*^(2)^(0); Indistinguishability, *M*; Extraction efficiency, CE; Repetition rate, RR; Brightness, ⟨*n*⟩; Operation temperature, OT; Spin coherence time, *T*_2_.

Application	*g*^(2)^(0)	*M*	CE	RR (GHz)	⟨n⟩	OT	*T*_2_ (ms)

quantum key distribution	<0.1	not critical	>0.5	>1	1	room temperature	–
optical quantum computation [80]	<0.01 or 0.001	>0.99	>0.99	1	1	not critical	–
spin–photon entanglement [64,82]	<0.1	>0.8	>0.3	>0.1	1	not critical	>1
quantum radiometry [83]	<0.1	not critical	>0.99	>0.9	1	not critical	–
imaging	not critical	not critical	>0.5	not needed high	not critical	room temperature	–
magnetic sensing	not critical	not critical	>0.5	not needed high	not critical	room temperature	>1

### h-BN optical point defects and SPSs

Hexagonal boron nitride (h-BN) is boron nitride’s most used polymorph. The electronic structure of h-BN has been studied by luminescence, as well as by other means, such as optical reflectance and absorption, electron energy loss spectroscopy, X-ray absorption, emission, and inelastic scattering. Regarding luminescence studies, PL is the light emission, i.e., the electromagnetic radiation from matter after the absorption of photons. It is originated by photons exciting electrons to a higher energy level in an atom. This photo-excitation is then followed by various relaxation processes. During these relaxation processes, other photons can be re-irradiated. One interesting parameter is the zero-phonon line (ZPL), which is the difference between the lowest values of the excited state and ground state. ZPL lines have distinct peaks in the experimental PL spectrum. The ZPL is the narrow component at a specific frequency of the absorption line of electronic excitation. The broader feature is then the phonon sideband (PSB). In h-BN, there are direct and indirect bandgaps. Bandgap energy values largely varying from 3.6 eV to 7.1 eV have been reported in the literature [84–86]. Theoretical calculations for the h-BN band structure also show significant differences in the eV values. Some density functional theory (DFT) in the local-density-approximation (LDA) computations suggests a lower indirect gap around 4 eV. Calculations with other methods suggest a higher value of about 5.95 eV [87]. In experiments, the variability of the properties is even larger. For example, stacking influences the electronic properties of h-BN [86]. The indirect bandgap is close to 4 eV for the dominant form of h-BN. A laser effect at λ = 215 nm in a monocrystalline sample under e-beam excitation observed with excitation at 262 nm was reported by Watanabe et al. [88]. Data about the h-BN PL is still scattered, despite the fact that a fine-structure luminescence considered to be inherent to the BN molecular layer in the range λ = 300–500 nm was already observed by Larach et al. [89] more than 60 years ago. Katzir et al. [90] observed a blue PL continuum in the range 390–450 nm (excitation at 320 nm) and attributed this phenomenon to deep levels of carbon impurities. The luminescent mechanism in h-BN is not yet fully clear. There is a large variability in the shape and position of PL spectra, which is interpreted based on impurities and sample preparation conditions [91–92]. Museur et al. [92] report on the effect of surface oxidation under UV-laser irradiation on PL in h-BN. Very likely, this spreading of shapes and positions possibly originates from the many relevant parameters that are not fully under control in the preparation of the material and the way the experiments are conducted.

In the first experiments, ensemble measurements using cathode-luminescence and optical spectroscopy in deep ultraviolet [91,93–94] were used for the characterization of stacking faults and point defects.

More recently an aberration-corrected high-resolution transmission electron microscopy technique has been used to resolve atomic defects in a freestanding single layer of h-BN with triangle shapes. The most commonly formed defects found are boron monovacancies [65] as the dominating zigzag-type edges of the defects are nitrogen terminated.

Individual defects with nanoscale resolution were isolated and manipulated by using scanning tunneling microscopy (STM) [95]. This has prompted the investigation of SP confocal microscopy to observe SPEs from various h-BN types of materials from bulk to monolayers, however with similar unclear attributions.

**Figure 2:**
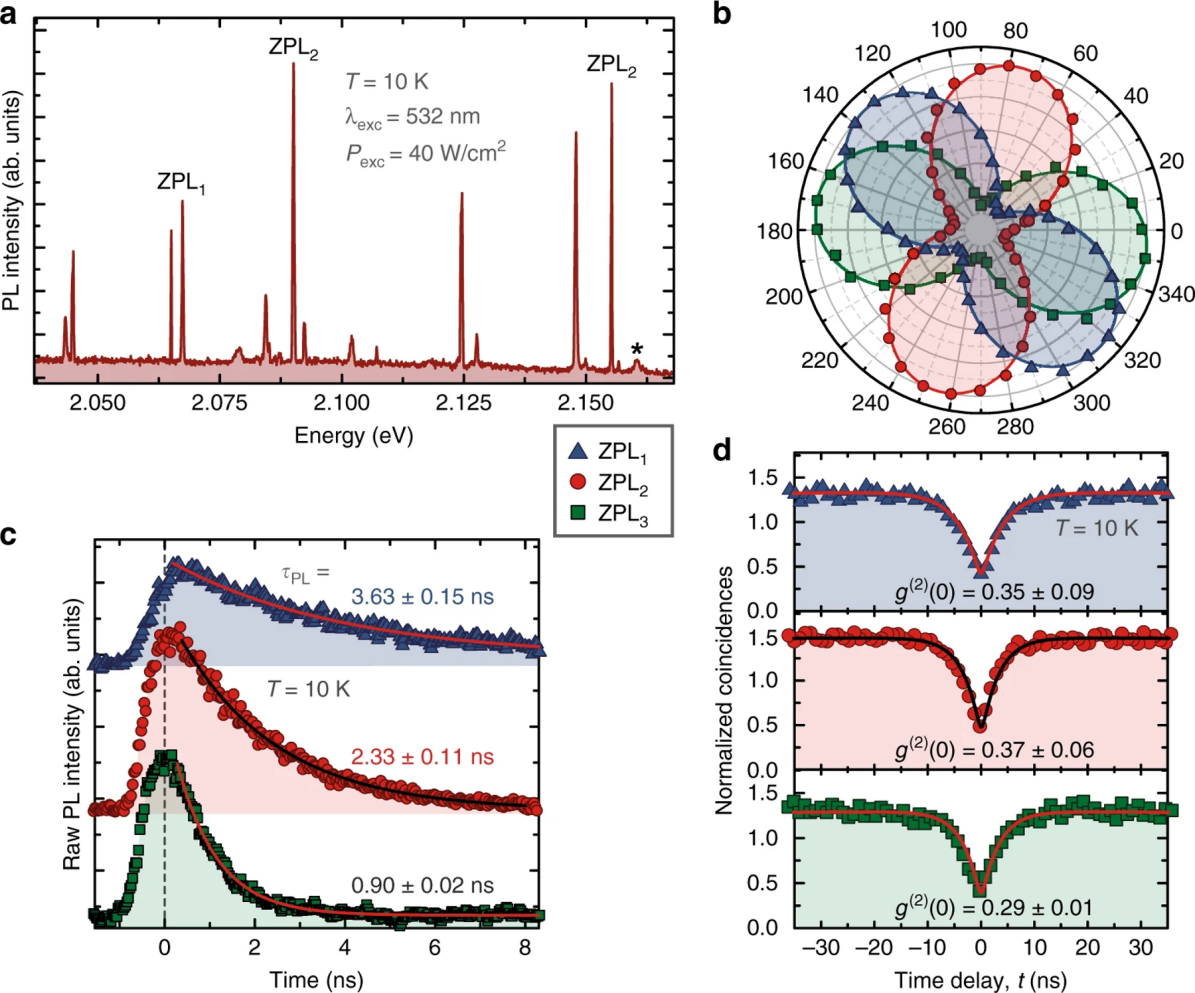
Example of h-BN SPE properties. (a) Micro-PL spectrum at 10 K excited with a 532 nm CW laser with a power density of ≈40 W/cm^2^. Multiple narrow ZPLs are labeled ZPL_1_, ZPL_2_, and ZPL_3_ and characterized in (b)–(d). Another peak indicated with an asterisk is a Raman peak of h-BN. (b) Normalized and background-corrected low-temperature polarization-dependent intensities of peaks ZPL_1_ (blue triangles), ZPL_2_ (red circles) and ZPL_3_ (green squares), showing SPEs with a high linear polarization degree. (c) PL lifetime associated with the three ZPLs in b). (d) Normalized *g*^(2)^(τ) function of the ZPLs in b and c. The experimental data are shown with their theoretical fits. For lifetime measurements a 442 nm pulsed laser is used rather than a 532 nm CW laser. Reprinted with permission from [103], copyright 2019 Springer Science and Business Media LLC. Article licensed under a Creative Commons Attribution 4.0 International License https://creativecommons.org/licenses/by/4.0/.

Despite the PL variability in h-BN, very recently, deep-level, atom-like luminescent defects in h-BN have been considered for non-classical SPE mostly in the 2 eV spectral region using intra-bandgap excitation [96]. These luminescent centers, which were extremely robust at high temperature, exhibit linearly polarized ultra-bright sources of anti-bunched light [44–45,48,97–100]. However, the standing of h-BN as an SPE is still thwarted by the wavelength variability of the ZPL from one emitter to another, which spans a broad spectral range from the UV to the visible up to the near-infrared regions [101–102]. As an example of some of the results, Figure 2 taken from [103] summarizes the photo-physics of some SPEs in h-BN. The µ-PL spectrum reported here, taken at low temperature, discloses multiple ZPLs originating from bright and optically photo-stable SPEs found within the excited h-BN grains. As they are narrow ZPLs frequently observed in h-BN layers, they were here attributed to the presence of point-like defects. Here the starting material is commercially available h-BN powder in the form of around 1 µm grains, where SPEs were induced by thermal treatment not further specified. These defects can confine electronic levels within the bandgap, acting as recombination centers, causing SP light emission. Lazić et al. [103] observed patterns of PL peaks spanning across a spectral band of ≈900 meV for each location on the sample. In this example, the PL peaks are centered at 2.067 (≈600 nm), 2.090 (593 nm) and 2.155 eV (575 nm), here labeled as ZPL_1_, ZPL_2_, and ZPL_3_, respectively.

In Table 3 a summary of the properties of SPEs found in various h-BN material types with various fabrication techniques is provided with related information of their optical or electron excitation, PL, operation temperature, and brightness at saturation. The description of these sources from the PL point of view, host material and fabrication methods are detailed in the following sections.

**Table 3:** Summary of SPEs in h-BN 2D (single and multi-layers) and bulk material.

h-BN	PL (CL) (nm)	Optical excitation (nm) [electron excitation (keV)]	*T* (K)	Brightness (kcts/s)	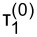 (ns)	Assignment/reference

single and multilayers	623	532	RT	4000	3	tentative antisite complex V_N_N_B_ [43], but also the V_N_C_B_ [104]
exfoliated multilayer flake monolayer CVD	569–697	532, 594	RT	20–25	2–3	tentative VB but other options are possible such as Stone−Wales defects [45]
Multilayer flakes	570–740	532	RT	>400	1.8–4.5	unassigned [44]
exfoliated, multilayers	(303)	[60]	150		1.1	C_N_ [97]
single crystal	596, 629	532	RT	4000	3.1	different charge states of the same defect [98].
single crystal	618, 629, 770–900	532, 675	RT	200	1	unassigned [100]
flake multilayers	565−775	532	RT	100–2400	2.9–6.7	[105]
flakemultilayers	639, 697	532	300–800	–	3.6	unassigned [106]
flakes	387–896	325–780	4–1100	2–16	1.12–1.35	variety of impurities such as CN, B, HN or VB, VN [107]
CVD h-BN few layers	580 ± 10	532	RT	1400	3	[108–109]
flakes	(435)	[2-10]	RT		2.6	ultra-pure material [110]

We will now discuss the discovery of these SPSs in the different material platforms of h-BN from bulk to 2D, 1D, and 0D material form.

### Bulk material

Martínez et al. [98] shows for the first time an SPE in bulk commercial h-BN single crystal. It appears that the SPE was found randomly in the material without treatment. Two families of spectra with ZPL energies at 629 nm and 596 nm were identified. Different charge states of the same defect were attributed to the two emissions, while the small variation of the ZPL energy in each group (±20 meV) was attributed to variations of the strain in the h-BN matrix, as well as photo-conversion to a dark state that may be responsible for the blinking. Only 5% of the emitters were photostable. The photo-dynamics indicates the presence of a metastable state (3-level system) with 1/τ_1_ = 0.51 ns^−1^, 1/τ_2_ = 0.14 μs^−1^, and *c* = 0.6. The optical transition lifetime is 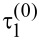 = 3.1 ns while the lifetime of the metastable state modeled with a linear pump dependence is 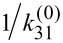 = 210 µs. A saturation count rate of a single emitter of ≈4000 kcts/s at 400 µW was observed, making it one of the brightest SPSs observed in any material at room temperature. Similarly, bulk BN was studied by [100] after annealing the sample in Ar at 850 °C for 30 min and at 0.5 Torr to increase the concentration of defects with similar ZPLs previously reported. However, they were found to photo-bleach with excitation at 675 nm, and emitters with ZPLs of 760 nm were found with a much shorter lifetime and a saturation count rate in the 200 kcts/s. The photo-dynamics of these emitters indicate the presence of a multilevel system with three metastable states with long decay rates of 480 ns, 5 µs and 31 ms. Blinking was observed and no assignment was provided. The results of the SPEs from h-BN bulk seem to indicate these are different emitters from thin layers as described in the following.

### Monolayer and multilayers/flakes

The first work showing SPE from multilayers and single-layer h-BN was published by Tran et al. [43]. Here commercial material was annealed in Ar to prevent oxidation and to achieve a higher density of emitters. An SPE PL at 623 nm was observed and tentatively assigned to the antisite point defect V_N_N_B_. The SPE is fully polarized along one direction for both excitation and emission, a relevant property for SPE. The SPE was modeled with a 3-level system with 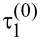 ≈ 2.2 ns, 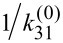 ≈ 2.2 ns and 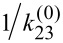 ≈ 67.9 ns.

**Figure 3:**
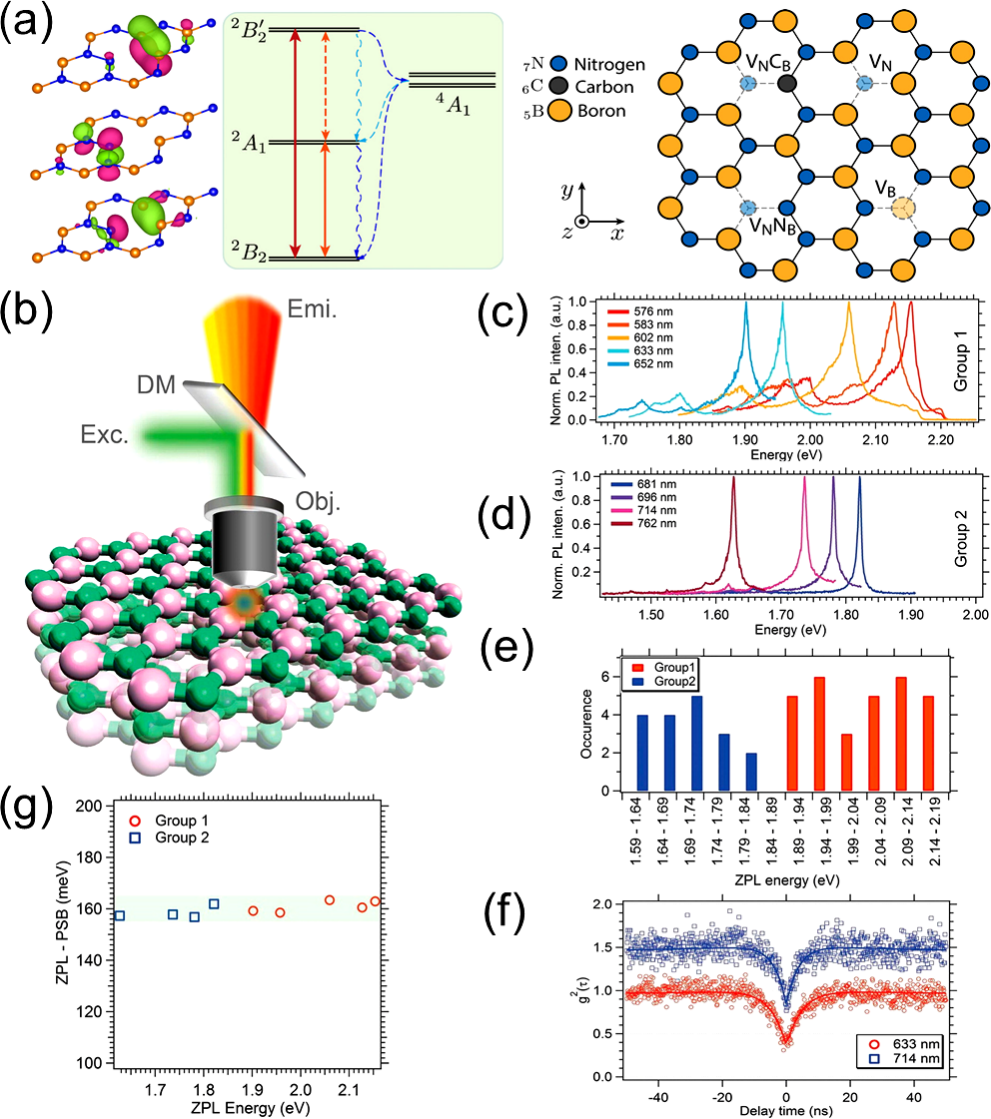
(a) Energy levels and orbitals of the ground state V_N_N_B_ (antisite) on the left. On the right, the atomistic geometry of some of the common defects in h-BN attributed to the SPEs. The V_N_N_B_ (antisite) and V_N_C_B_ defects have C_2v_ point group symmetry with their axis of symmetry (*x*-axis here) laying in the plane, while the monovacancy V_B_ and V_N_ have D_3h_ point group symmetry with the symmetry axis (*z*-axis) pointing out of the plane. Images reprinted (adapted) with permission from [111], copyright (2018) American Chemical Society. Multi-emission from point defect SPSs in h-BN, measured using a PL setup described in (b): Obj, the objective lens, DM, a dichroic mirror, Exc, the excitation source, Emi, the collected photons. Exc is at room temperature using a 300 μW CW 532 nm laser. Examples of ZPLs of SPEs are named in Group 1 (c) with ZPLs at 576 nm (2.15 eV), 583 nm (2.13 eV), 602 nm (2.06 eV), 633 nm (1.96 eV), and 652 nm (1.90 eV). Group 2 in (d) have ZPLs at 681 nm (1.82 eV), 696 nm (1.78 eV), 714 nm (1.74 eV), and 762 nm (1.63 eV). (e) ZPLs for numerous SPEs in the above groups are represented in a histogram. (f) *g*^2^(τ) functions acquired using an acquisition time of 20 s with zero delay at 0.39 and 0.34, respectively. (g) The difference in the energy of the ZPLs and PSB versus ZPL energy. Images reprinted (adapted) with permission from [105], copyright (2016) American Chemical Society.

Ab-initio simulations have confirmed that V_N_N_B_ is likely the color center associated with the SPE [111] (Figure 3), and this defect has been modeled as a candidate to provide optical spin readout [112] and is thus a candidate for a spin qubit. Other modeling based on DFT and constrained DFT attributes the SPE to a V_N_C_B_ defect [104].

In [97] few layers of exfoliated h-BN were studied under cathode-luminescence (CL) combined with a Hanbury Brown and Twiss interferometer to identify their SPE in the UV (303 nm) with certain native point defect origin due to the high localization observed using CL maps. The point defects were likely carbon substitutional at nitrogen sites (C_N_). Another broad emission not associated with any SPE was present in the same spectral region, here attributed to intrinsic defects related to electron irradiation.

After these experiments, the hybrid density functional was used to simulate common as-grown vacancy and antisite defect properties demonstrating that they require high formation energies. Thus it is unlikely they could form from the typical growth conditions under thermodynamic equilibrium [113]. This work seemed to rule out previous assignments given to the first SPE in h-BN and rather identify interstitials or their complexes as more possible centers. By simulating substitutional carbon and oxygen, interstitial hydrogen and boron vacancy–hydrogen complexes, it is shown they are low-energy formation defects in h-BN. This suggests that the assignment is presently controversial also considering the successive verification of SPEs as discussed in the following.

In [45] h-BN exfoliated flakes, monolayer chemical vapor deposition (CVD) and in-house h-BN were studied. Here the correlations between material structural features and the location of SPEs from bulk down to the monolayer was studied at room temperature. Chemical etching and ion irradiation are used to generate the SPEs in h-BN various materials. Their photo-dynamics analysis reveals miscellaneous transition rates for the optical and non-radiative transitions of the emitters. The SPEs show a very good photo-stability under ambient conditions and in monolayers. A correlation between the defect orientation and the h-BN hexagonal structure is observed by studying the excitation polarization between different SPEs. A large variety of SPEs was observed. It was observed that thinner and edgier flakes had higher SPE density, and in the CVD material, SPEs had more brightness compared to flakes. The SPEs could be modeled by 2-level and 3-level systems with a variety of decay rates: for a two-level system emitter with ZPL at 696 nm, 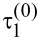 = (0.77 ± 0.14) ns was found; for a 3-level system SPE with ZPL at 580 nm excited state relaxation rate 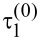 = (3.29 ± 0.11) ns, a 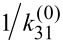 = (6.8 ± 3.5) ms, and an ISC rate of 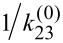 = (0.33 ± 0.03) ms; in the monolayer SPEs with ZPLs 660, 657, 630, and 637 nm the ISC transition rates were very different from the flakes with 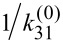 = (0.395 ± 0.065), (1.7 ± 0.3), (0.47 ± 0.05), (0.8 ± 0.2) ms and an ISC rate of 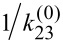 = (0.34 ± 0.22), (1.9 ± 1.4), (0.22 ± 0.07), (0.38 ± 0.16) ms.

Multicolor emission from yellow to far-red was also found in h-BN flakes under different fabrication procedures (Figure 3) [105]. Kianinia et al. [106] shows that SPEs in h-BN flakes can operate up to 800 K, constituting a robust SPS compatible with device fabrication temperature procedures.

In [107] SPEs from h-BN flakes were observed with ZPLs ranging from 357 to 896 nm using different excitation wavelengths from 442 nm to 780 nm with operation from cryogenic to very high temperatures (1100 K). DFT was used to understand the origin of such broadband emission possible from defect states related to H, O, C, N, and B induced defects in bulk and monolayers indicating the emission-related defects population. However, the PL is not sufficient to univocally identify the type of defects, while the correlation with material properties and SPEs should be performed.

In [114] h-BN the quantum emission was correlated with the material’s local strain using a combination of PL, nanobeam electron diffraction, scanning transmission electron microscopy (STEM) and CL. ZPLs in PL and CL were observed ranging from 540–720 nm. Four distinct defect classes were attributed to the observed emission range. One defect class with ZPL emission centered at 580 nm had PL and CL matching with spectral variability due to strain. This is different from another defect class with mostly matched CL-PL ZPLs near 615 nm. A third defect class at 650 nm has longer wavelength CL emission, and a fourth defect centered at 705 nm has a small shift between its CL and PL peaks of ≈10 nm. The large spectral variability of SPEs cannot be attributed solely to strain, and strain alone is not required to activate the emission. Additionally, not all defects were found at the edges of the flakes, as previously found. While high-temperature operation indicates very robust emitters, the emission wavelength of the fluorescent defects in h-BN is uncontrolled, with widespread ZPLs from UV to NIR, which limits the potential development of h-BN-based devices and applications and their controllable formation. In [109], it is shown that in CVD large-area, few-layer h-BN films, more than 85% of the emitters have a ZPL at (580 ± 10) nm, while maintaining a high density of emitters. Such methods based on high-temperature annealing in air and ultraviolet ozone processing are effectively used to improve SP purity (*g*^(2)^(0) ≤ 0.1) and the linewidth (FWHM room temperature of ≈3 nm) of the ZPL of CVD-grown h-BN [108].

By control of the boron diffusion through copper during atmospheric pressure CVD, a method known as a gettering technique is developed to control the material crystal purity. The resulting SPE ZPLs are more frequently placed between the regions 550 and 600 nm or from 600 to 650 nm. The results can improve the understanding of quantum emitter formation in h-BN [115].

### Nanotubes and nanococoons

Another class of 1D and 0D h-BN nanomaterials, such as nanotubes (BNNTs) and nanococoons, were found to exhibit SPE. BNNT can be associated with 2D h-BN hexagonal sheets rolled into a closed nanotube structure, making them a type of 1D material up to 200 µm long. Here the SPE is attributed to effects similar to SPEs from TMC flakes except that BNNT has a large bandgap (5.95 eV) that is not affected by the geometry and their operation is at room temperature. When the BNNT diameter increases, the material can be assimilated into a 2D material, and as such, it can be considered a hybrid 1D and 2D material.

In [116], commercially available BNNTs were fabricated using a catalyst-free high-temperature pressure method, and the laser heating method was studied in terms of SPEs. Non-treated BNNTs provided photostable SPEs down to the single nanotube, either in dispersed or suspended material. The SPEs are combined with high-resolution SEM to categorize emission down to a material scale of less than 20 nm. The SPEs are characterized by 3-level systems with a transition rate similar to the ones observed in h-BN flakes (*k*_21_^0^ = 227 MHz, *k*_31_^0^ = 529 MHz, and *k*_23_ = 127 MHz), albeit some emitters had an optical lifetime five times longer due to the fact that their dimension was much smaller than the excitation wavelength (532 or 594 nm), exhibiting an antenna effect. By artificially curving h-BN flakes by using strained BNNTs on diamond and zirconia pillars, similar SPE spectral features (PL in the region from 630 nm to 680 nm) were observed. In general the SPEs from this material were of poor purity and large variability of the lifetime. In [117] SPEs from point defects in BNNTs, with PL at 571 nm and 569 nm, an average diameter of 50 nm was shown to exhibit optical modulation of the fluorescence by exciting with a 1064 nm laser that populated a dark state, followed by decay to the ground state. The excited-state lifetime of 5.6 ns and the second photon correlation decay time of 250 ns and 321 ns provide a NIR modulation of 10–20%. Due to the larger size of the BNNTs, the PL is photostable, althougt the SPE purity is still not exceptional. In [118] SPE in a ball-like 0D BN allotrope with dimensions ≈1–100 nm, known as nanococoon BNNC, is shown. The density of the SPEs was increased by dual-beam focused ion beam and SEM to selectively irradiate the sample with 10 keV gallium ions at a dose of 10^−14^ C/µm^2^ with subsequent annealing with argon at 1 Torr and 750 °C for 30 min. The lifetime, brightness, and PL stability of this SPE are similar to those in 2D h-BN, however with a wavelength variation smaller by a factor of five as compared to the SPEs in 2D h-BN. In addition, they also exhibited general lower brightness. All these SPEs in BNNTs and BNNCs operate at room temperature and as such provide an alternative platform for hybrid nanophotonics due to their small size, which can increase their emission coupling with plasmonics and photonics hybrid devices.

### Fabrication and localization methods

Here we summarize the large variety of fabrication methods used to create SPEs in various types of h-BN materials (mostly flakes and CVD h-BN), in the attempt to determine their physical origin or atomistic attribution. The methods do not provide information (except in strain-induced formation and neutron irradiation) of the physical origin, however, the large variety of effective methods indicates multiple origin and facile fabrication, albeit only two methods can be considered quasi-deterministic.

**Figure 4:**
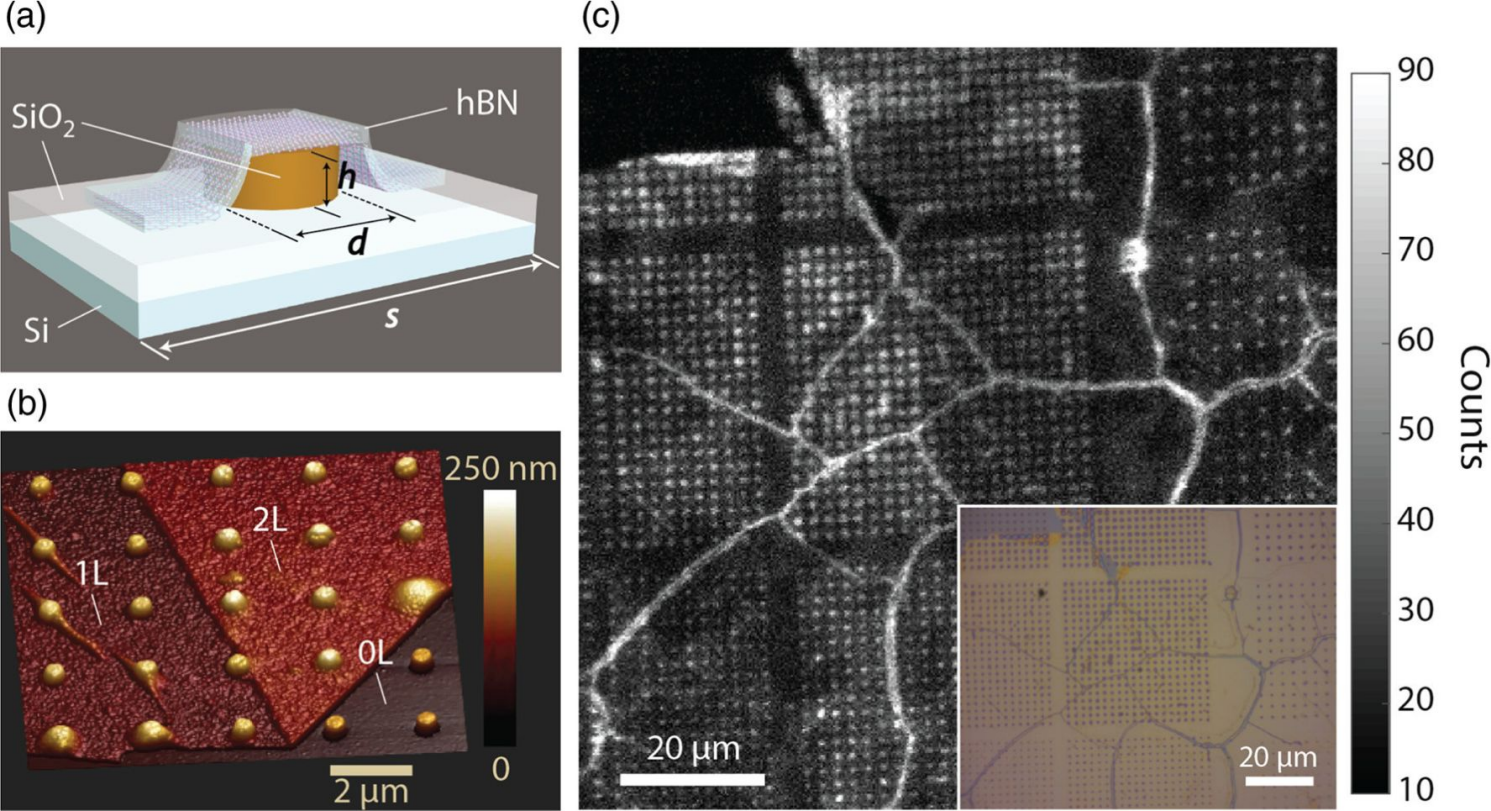
h-BN SPE strain-induced fabrication. (a) Schematics of a ≈20 nm-thick flake of h-BN on a nanopillar fabricated on a silica substrate. The nanopillars were fabricated by electron beam lithography and sized with variable height *h*, pitch *s* and diameter *d*. (b) 3D rendering of an AFM image of the h-BN flake showing 1 and 2 layers (1L and 2L) on bare silica nanopillars (0L). (c) The confocal image at room temperature (main) and optical microscope images of the nanopillars (inset) with *s* = 2 μm (left and center arrays) and *s* = 3 μm (far right). The nanopillar height is 155 nm, while the diameter varies from 250 nm to 500 nm with an increment of 50 nm from the lower left-hand array to the top center array. Reprinted (adapted) with permission from [124], copyright 2018 Optical Society of America under the terms of the OSA Open Access Publishing Agreement.

A number of different methods have been used: annealing [105,119] up to high temperatures and in various environments, with an increased number of defects observed with increased temperature; ion beams of various types, such as Si, O, B, boron−nitrogen (BN) complexes [119], He and N at low fluences [45]; chemical etching [45] based on the use of peroxymonosulfuric acid (H_2_O_2_:H_2_SO_4_) and with an additional step of phosphoric and sulfuric acid (H_2_SO_4_:H_3_PO_4_), where this second method was found to increase the estimated density of the SPEs from 0.09 to 0.54/µm^2^, indicating that thinner and edgier flakes provide higher SPE density; electron irradiation [105,119–120] from low to high energy electrons, with the high energy electrons improving the yield and the spatial distribution of the emitters away from the edges in the center of the flake; oxygen plasma etching associated with annealing [121] and in particular a process of only two steps, including Ar plasma etching and subsequent annealing in Ar, yielding a considerable increase in the concentration of emitters in h-BN [122]; laser irradiation [123] even if the formation origin, in this case, is unclear; substrate strain-induced where a 20 nm-thick h-BN film grown via CVD is transferred into SiO_2_ nanopillars on Si substrates [124]. In this case, the SPEs are activated due to potential wells induced by material deformation trapping carrier, localized near the points where the h-BN film reaches the highest curvature (see Figure 4). Here the emission has PL mostly in the 540 nm region with more controlled formation and properties. Random generation in the material using bubble strain-induced SPEs is also demonstrated in [125].

Focused ion beam irradiation was used to mill holes in the h-BN to achieve array-like SPEs around the perimeter of the holes [126]. This method yield is very high compared to the nanopillar substrate method, and SPEs have a similar PL distribution of the strain-induced emitters and brightness comparable to the brightest SPEs found in h-BN.

Neutron irradiation [127] shows a homogenous distribution of emitters within the 2D h-BN multilayers and the density of induced color centers is positively correlated with neutron fluence. This approach suggests that the atomic origin of the color centers emitting at 580 nm is the V_B3N1_ and it is a viable method to achieve an ensemble of SPEs. SPEs in 2D materials have proved to be resistant to gamma-ray irradiation [128]. We can tentatively assign a larger variability of ZPLs in methods involving annealing and chemical etching (see Table 4), while methods like electron irradiation, ion irradiation, and neutron irradiation tend to have emission more likely in the region of 580 nm if annealing is not performed. Electron and ion irradiation seems to yield two different groups of emitters from the ZPL’s point of view [105]. Using strain induced formation, the emission appears shifted to shorter ZPLs at around 540 nm. However, a dependence on the material type also influences the ZPL’s variability based on the observed difference between multilayer flakes and CVD monolayers, where the former has a larger observed density of defects and ZPLs.

CVD monolayer h-BN SPEs were studied in [45] after treatment and their ZPLs were reported in the range of 583–691 nm. Using low-pressure CVD [108–109], large-area, few-layer h-BN films can be grown on copper, nickel and iron substrates, with a high density of SPEs of ≈100−200 per 10 × 10 µm^2^ with more defined ZPLs at (580 ± 10) nm. The most promising approaches seem to be the strain-induced methods and the focused ion beam method, possibly combined with low-pressure CVD for more controlled material quality.

In Table 4 we summarize the main methods used for the creation of the SPEs observed so far.

**Table 4:** Methods for fabrication of SPEs in h-BN and a comparison of ZPLs.

Methods	Electron irradiation	Neutron irradiation	Ion beam methods	Substrate strain-induced	fs-laser writing	Focused ion beam methods	Annealing/chemical and plasma etching

energy	15 keV [119] 2 MeV [120]	1.2 eV [127]	(B, O, Si, BN) 50 keV [119] low doses of He, N [45]	nanopillars less than 150 nm high and pitched 2 µm [124]	140 fs laser [123]	Ga 5–30 keV [126]	850 °C, 30 min in Ar [119] 200–1200 °C, 30 min, H, O, ammonia [105] (H_2_O_2_:H_2_SO_4_) and (H_2_SO_4_:H_3_PO_4_) [45], oxygen plasma and annealing [121–122,129]
fluence	5 × 10^18^ e/cm^−2^ 1 × 10^15^ electrons/cm^−2^	1.5–2.5 10^13^ and 10^14^ neutrons/cm^−2^	10^10^ cm^−2^		multiple laser pulses 80 MHz	10^−13^–10^−13^ C/µm^−2^	NA
yield	higher with MeV electrons	–	marginal increase of SPEs or just increased stability	all nanopillars show emission	very low	31%	high density
ZPLs	580 nm and second peak at 623 nm; 580–600 nm, with second peak at ≈644 nm [120]	570–592 nm, central most likely 580 nm	600 nm and second peak at 650 nm [119] and 569–697 nm [45]	mostly at ≈540 nm, with sideband peak at 588 nm (range from 530–610 nm)	≈630 nm	540–620 nm (most common 540 nm)	565−775 nm [105,122] 569–697 nm dominant peak at 580 nm [45]
array singles	no	no	no	yes	no	yes	no
comments	SPEs found at the edges of flakes for low energy while high energy also in the center of flakes and more homogeneously distributed along with multiple layers [129].	ensemble concentration scales with fluence	SPEs found at the edges of flakes	SPEs at the nanopillar edges	SPEs near the ablation area, unclear origin formation	500 nm diameter circular holes with a center-to-center separation of 1 µm in each region.	SPEs are stable even after annealing in harsh gaseous environments. SPEs found at the edges of the flakes and grain boundaries, as well as in top layers of flakes near the surface [129].

### Spectral study and control of SPSs

Showing strong brightness with a narrow linewidth and a high Debye–Waller factor even at room temperature, defects in h-BN have the strong potential to be used for practical SPEs. As such SPEs in h-BN have been studied from the point of view of spectral line width, ZPLs at low-temperature distribution, and spectral diffusion by several groups. Since the robustness to temperature is crucial for their practical application, and also the temperature characteristics are strongly related to the luminescence mechanism and the interaction to phonons, the effect of temperature on SPEs should be clarified. Further, spectral studies of SPEs and their coherent control are essential to establish a spin–photon interface for future quantum networks. In this respect, spectral instabilities common in solid-state emitters can hinder many applications.

A strong temperature dependence of ZPLs was first observed, as shown in [44,106], in multilayer h-BN flakes. Specifically, Jungwirth et al. [44] investigated two different SPEs in h-BN flakes emitting at 575 nm and 682 nm and clarified their temperature dependence of the ZPL peak shift, line width, and PL intensity ranging from 4 to 300 K.

The temperature-dependent line width, spectral energy shift, and intensity differing ZPLs are described by a lattice vibration model that considers piezoelectric coupling to in-plane phonons. The temperature dependence can result in spectral instability and diffusion. They found that the line width of ZPLs was wider than their natural linewidth, suggesting that the broadening of the line width is attributed to the phonon-mediated mechanism. Kianinia et al. [106] demonstrated stable single-photon emissions over a long time (more than 60 s) from two different SPEs in h-BN at 1.94 eV and 1.75 eV ranging from 300 to 800 K without any blinking or photo-bleaching. ZPL redshift due to the electron–lattice interaction and the broadening of emission linewidth due to the interactions with lattice phonons appeared, while the *g*^(2)^(0) values and the emission lifetimes were unchanged up to 800 K. The decrease in emission intensity accompanied by heating was caused by the increase in nonradiative transition rate, and the activation energies for 1.94 eV and 1.75 eV emitters were revealed to be 0.25 eV and 0.17 eV, respectively. The antibunching characteristics of SPEs were well fitted by a three-level model and the temperature dependence on emission intensity was explained by an additional energy level that contributed to the nonradiative transition. These results suggest new possibilities to integrate hBN SPSs with large-scale, on-chip quantum photonic devices that work under ambient conditions or elevated temperatures.

Resonant excitation of SPEs in h-BN is also used to control their quantum properties such as spectral broadening and Rabi oscillation.

Using resonance excitation with different excitation wavelengths (PLE spectroscopy), a large variety of defect emissions in the UV–NIR (357–896 nm) have been found at room temperature in flakes [107]. Different emission peaks have different resonant excitation wavelengths. Tan et al. [107] have revealed that, for instance, SPEs emitting at 685 nm and 767 nm showed two resonant excitation wavelengths of 494 nm and 528 nm. DFT calculations suggest that the wide range of PL emission from UV to NIR is attributed to different types of defect structures. However, the single-photon emission from UV and NIR peaks has not been demonstrated and thus further investigations are necessary to explore SPEs in h-BN. The ZPL spectral fluctuation (diffusion) is often found due to ionization and the charging of neighboring defects leading to the Stark shift. SPEs in h-BN flakes exposed to blue (405 nm) laser light show pronounced fluorescence instability taking the form of large ZPL spectral diffusion and discrete jumps of up to 100 nm (red-shifted) under ambient conditions. However, they are most stable under illumination at 532 nm [130]. A photochemical reaction with activation energy between 2.3 and 3.0 eV potentially contributes to the ZPL discrete jumps.

An SPS with Fourier transform (FT) limited linewidth is required for applications in optical quantum computing and spin–photon entanglement distribution. FT limited linewidths down to ≈55 MHz have been observed from a ZPL line of SPE in h-BN [131–132]. Further study of on-resonant excitation at room and low temperature was performed, confirming similar results of spectral linewidths of h-BN SPEs in flakes narrower than 1 GHz with an average spectral diffusion time of around 100 ms [133]. h-BN flakes were investigated at liquid helium temperature and using off and on resonance excitation. More than 600 SPE spectral linewidths were determined [131–132]. In addition to the variability of the ZPLs (from 580 nm to 800 nm), the spectral linewidth is also variable from a minimum of 70 GHz to a maximum of 400 GHz at 5 K, with considerable spectral instability and diffusion. Broadening of the ZPLs was reduced after annealing and with on-resonance excitation and only two SPEs were found to have a 55 MHz line width corresponding to a natural linewidth of the transition with a lifetime of 2.88 ns. In one case, a 64 MHz linewidth persisted up to room temperature [132].

About 20% of PLE scans show the FT limited lines with a homogeneous linewidth of (46–60) ± 10 MHz. Moreover, the FT limited line width was constant across the temperature range from 3 to 300 K within the measurement uncertainty of the FT limit [131–132]. This temperature stability can be explained by the lack of coupling to low-frequency acoustic phonons (or any other dephasing mechanism) on the timescale of the laser scans. The center’s decoupling from phonons is a fundamental consequence of the material’s low dimensionality.

In [134] resonant excitation measurements also confirmed SPEs in h-BN with strong spectral diffusion corresponding to a linewidth broadening of 0.6 GHz and a time scale of 30 ms in the weak power limit. In the limit of high power excitation, *g*^(2)^(0) measurements revealed coherent optical Rabi oscillations. This is an example of coherence control of optical transitions in h-BN flakes at low temperature.

**Figure 5:**
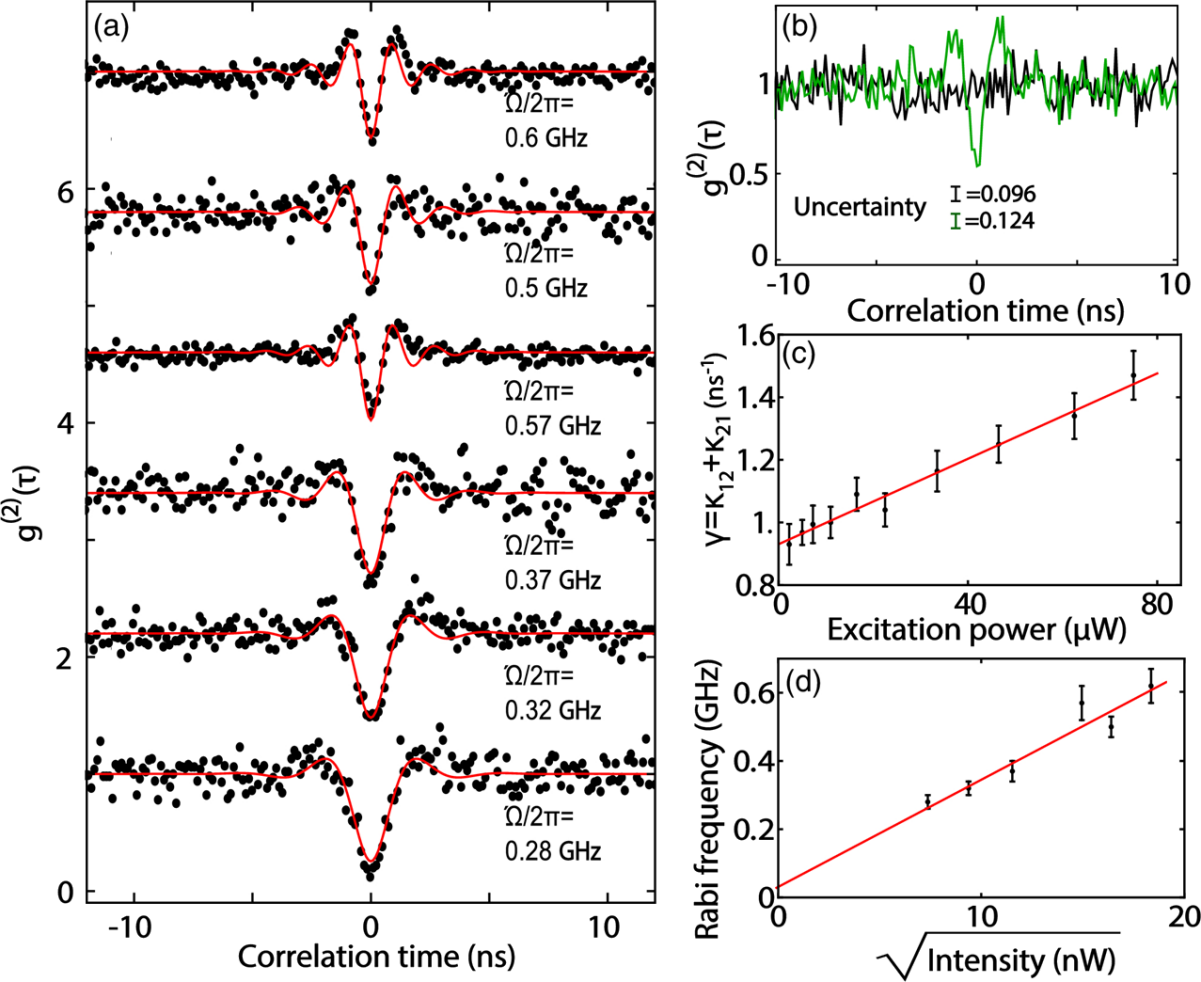
Rabi oscillations. (a) *g*^(2)^(τ) function measured using the phonon sideband (PSB) photons as a function of excitation power. Traces are vertically shifted for clarity. (b) Comparison of *g*^(2)^(τ) for PSB photons (green) and white light with the same average count rate (black). The indicated uncertainties are standard deviations of the two curves. (c) Decay rates (1/τ_1_ in Equation 21) extracted from nonresonant *g*^(2)^(τ) measurements versus excitation power. The solid line is a linear fit to the data to estimate the spontaneous decay rate of the emitter. (d) Estimated Rabi frequency from (a) as a function of the square root of the laser intensity. Figure reproduced with permission from [134], which is an article licensed under a Creative Commons Attribution 4.0 International License https://creativecommons.org/licenses/by/4.0/.

In such a measurement scheme (PLE spectroscopy), the coherent population cycling between the ground and the excited states of SPE provokes an oscillation structure in the second-order correlation function *g*^(2)^(τ) governed by the characteristic frequency, i.e., Rabi frequency, since the oscillation is imprinted into the phonon-assisted fluorescence [134]. The Rabi frequency (Ω) is proportional to the electric field amplitude associated with the excitation laser power and the *g*^(2)^(τ) function changes according the Rabi frequency (Figure 5a). One can identify the first peak of an oscillatory signal and its linewidth narrowing when the excitation power is increased. This is an important step toward utilizing coherent optical control in 2D materials for the realization of scalable quantum information processing.

Electrical control of the ZPL spectral emission (Stark tuning) can be applied to SPEs in h-BN flakes and h-BN CVD, as studied by several groups. The electrical control of the emission is important to tune emission to the photonic cavity.

Stark tuning is obtained by applying an external electric field. The Stark tuning of SPEs in few-layer h-BN flakes in addition to thick h-BN flakes has been demonstrated at 10–300 K [135–138]. The first demonstration of Stark tuning of SPEs in h-BN was achieved by Noh et al. [135] to our best knowledge. They fabricated graphene electrodes on a 100–200 nm thick h-BN flake and an applied electric field vertically, and they observed 5.4 nm per GV/m (52 meV per V/nm) Stark shift of ZPL (678 nm emission peak) at 10 K. They have also demonstrated the Stark shift at room temperature. The appearance of different types of Stark shifts implies that there are different types of SPEs in h-BN. Similarly, the 24 meV per V/nm Stark shift of SPEs in few layer h-BN with graphene electrodes was demonstrated at 15 K by Scavuzzo et al. [137]. The electric field also induced modulation of the emission intensity and the fluorescence lifetime [137]. The overall behavior of different types of Stark shifts can be well explained by a model involving different rates for electron and hole tunneling between the h-BN and graphene layers. They have also demonstrated the repeatability (reproducibility) of Stark tuning by sweeping applied electric fields. Nikolay et al. [136] has employed an atomic force microscope (AFM) with a conductive tip to an SPE at 670 nm which was sandwiched between the tip and an indium-tin-oxide-coated glass slide. This technology enables the application of a high electric field arbitrarily to h-BN nanoflakes. As a result, a very large Stark shift of 5.5 ± 0.3 nm (15.4 ± 0.8 meV) was realized at room temperature by vertically applying just 20 V. Xia et al. [138] fabricated multiple gold electrodes on an h-BN nanoflake to control the horizontal direction of applied electric fields and a giant room-temperature Stark shift of up to 43 meV/(V/nm) was observed. The Stark shift depended on the horizontal orientation angle of the applied electric field, showing the linear symmetry which was coincident with the polarization of emission intensity. The Stark shift as a function of the angle of the local electric field is well-fitted with an electric permanent dipole moment model based on perturbation theory to the first order.

Other methods have been demonstrated to be useful for the spectral tunability of SPEs in h-BN flakes, such as strain and acoustic-mechanical control. In [99] a method that uses strain control allows spectral tunability of h-BN SPEs of over 6 meV with improved material purity (*g*^(2)^(0) = 0.017) and a highest SPE saturation count rate of 7 × 10^6^ cts/s. Here the h-BN flakes were treated using a focused ion beam and annealed and then moved to a bendable beam to apply controllable strain. h-BN flakes irradiated by He ions were transferred on a bendable polycarbonate beam to controllably apply tensile and compressive strains. The ZPL shift due to a change in strain was also found by applying pressure.

Xue et al. [139] achieved a ZPL shift of 15 meV/GPa at a maximum at 20 K by using a diamond anvil cell (DAC) device. The device was developed by combining a classical DAC with a piezoelectric transducer (PZT) and the PZT-driven device can continuously generate pressure between approximately 0.4 and 4 GPa for the samples at low temperature. The pressure-dependent PL emission lines present three different types of pressure responses. The pressure coefficient of PL emission energy may be negative (redshift), positive (blueshift), or change the sign from negative to positive (redshift to blueshift). These behaviors can be explained by competition between the intralayer and interlayer interaction contribution, according to DFT calculations.

An alternative method to achieve spectral tuning can be based on the use of acoustic-mechanical effects induced by radio frequency (RF) surface acoustic waves (SAW) [103,140]. h-BN flakes were transferred onto a lithium niobate (LiNbO_3_) crystal with two interdigital transducers. The use of LiNbO_3_ provides wirelessly and non-destructively strong strain along the SAW propagation direction and perpendicular to the substrate surface. The SAW-induced hydrostatic strain is transferred from LiNbO_3_ to the h-BN flakes because of their high elastic response and increases with increasing SAW amplitude, resulting in the ZPL tuning of SPEs in h-BN. In [103] SAWs were applied to linearly polarized SPEs in the h-BN grains. The ZPL wavelengths and their emission time can be simultaneously controlled in situ by the strain induced by the propagating acoustic waves. The SAW-mediated energy shift of the defect emission in h-BN is of the order of 50 meV/% of strain at 10 K [103]. This dynamically tuned photon emission can be combined with the real-time-gating functionality for the production of spectrally and temporally identical SPEs. It should be noted that only a few percent of radiative defects were efficiently coupled to the SAWs [103].

Similar findings have been achieved by Iikawa et al. [140]. The SAWs modulate the intensity of the emission lines with a variation of up to 50% and oscillations of the emission ZPLs with an amplitude of almost 1 meV. It was shown that the dynamic piezoelectric field of the SAW also stabilized the optical properties of the SPEs. Moreover, they found that the presence of the SAW fields suppressed the spectral fluctuations caused by nearby shallow charge traps, leading to a more stable optical emission of the SPEs. Note that the contribution of the SAW piezoelectric field to the observed modulation was negligible.

The coupling between an embedded SPE and the vibrational (mechanical) modes of the hosting h-BN membrane via dispersive force holds the promise of further SPE tuning [141].

### Spin–photon interface

Zero field splitting (ZFS) results from the presence of more than one unpaired electron of a defect in solid and molecules undergoing various interactions, provoking their energy level splitting. Due to unpaired electrons at the defect site, the mutual interaction of the electrons can produce two or more energy levels even in the absence of applied fields. The ZFS is the measure of this degeneracy lifting and is responsible for many effects related to the magnetic properties of materials. This is observed in their electron paramagnetic resonance (EPR) spectra and magnetism study [142]. EPR studies in large bandgap semiconductors defects have been the object of research in the last 30 years. Studies on magnetic resonance methods in semiconductors can be found in [143].

ZFS for a defect in solid can occur both at the ground and excited state of their optical transition, as such it is a measurable energy difference. The most common cause for ZFS is a spin three-level system, which corresponds to a total spin *S* = 1 system. In the presence of a magnetic field, the levels with different values of magnetic spin quantum number (*m*_S_ = 0, ±1) are separated by the Zeeman splitting representing their energy separation.

The corresponding Hamiltonian is written as:

[25]
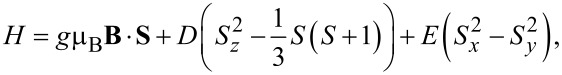


where µ_B_ is the Bohr magneton, *g* is the Landé factor, *S* is the total spin quantum number, *S*_x,y,z_ are the spin matrices, and **B** is the applied magnetic field. The value of the ZFS parameters is usually defined via the *D* and *E* Hamiltonian parameters. *D* describes the axial component of the magnetic dipole–dipole interaction, and *E* the transversal component. Values of *D* have been obtained for a wide number of materials by EPR measurements that provide more accurate data, while these values may be measured by other magnetometry techniques.

Point defects with electronic states inside the bandgap can be highly localized with wavefunctions confined to the atomic scale, exhibiting the strong exchange interaction necessary for spin-dependent relaxation channels.

Another technique such as optically detected magnetic resonance (ODMR), a double excitation method combining EPR with measurements such as fluorescence, phosphorescence, and absorption, can also be used to determine the ZPF. Here the sensitivity can reach a single molecule or single defect levels in solids like diamond [144] or silicon carbide [20,22].

EPR studies in h-BN date back to the 1970s [90]. One recent demonstration of the EPR signature of point defects in neutron-irradiated hexagonal boron nitride is shown in [145] for commercial h-BN powder with size of ≈70 nm. A zero-field splitting *D* = 1.2 GHz was associated with a broad visible optical absorption band (490 nm) and a near-infrared PL band centered at ≈820 nm. Here the EPR signal was tentatively assigned to point defects associated with doubly occupied nitrogen vacancies with *S* = 1. Their EPR signal intensities were strongly affected by thermal treatments when the temperature annealing was changed from 600 °C and 800 °C. SPE for this emitter was not confirmed.

Standard magnetic resonance is by far not sensitive enough to measure spin signals in 2D materials.

A step forward, which will significantly extend the functionality of h-BN SPEs for quantum applications, is to interface their optical properties with spin transitions and realize spin-polarization and optical spin readout schemes. The concept of the spin–photon interface has been extensively studied in the NV center in diamond [12]. The basic principle is that the triplet spin ground state of the defect can be polarized, manipulated and optically read-out owing to the spin-dependent excitation, decay and intersystem crossing pathways available to the system during the optical excitation-recombination cycle.

Requirements for systematic spin–photon interface point defects have been discussed in the literature. The identification and prediction of spin qubits or point defects with optical spin readout appears to be a complex task, and certainly, a need for precise computational approaches is now emerging as a necessary tool to tailor quantum spin qubits [146]. First-principles calculations are the most commonly applied method for fast investigation of point defect properties such as calculating the defect spin states and their basic optical properties. The evaluation of the real potential of candidate paramagnetic point defects for qubit applications is based on methods detailed in this paper [146]. However, for a systematic point defect qubit search, appropriate highly automated search algorithms are yet to be developed.

While the hypothetical spin defects in BN are similar to NV in diamond or NV and DV in SiC, they have been computationally studied in isotopically pure h-BN material [147], promising an even longer coherence time *T*_2_ (up to 30 ms) compared to 3D materials. However, their experimental verification is still not fully achieved as the isotopic purification of 2D material synthesis appears quite far from current material synthesis capabilities. Several initial studies have provided controversial results on the existence of magnetic properties of SPEs in h-BN [102,148]. Nevertheless, some promises of h-BN spin point defects are now emerging.

Currently contradicting or alternative models for the origin of defects aim to account for measurement differences in SPE properties in h-BN, while multiple origins likely play a role. The SPEs of h-BN largely exhibit an optical absorption and emission which is linearly polarized, indicating an optical dipole along the plane containing the axial symmetry. Based on these symmetry considerations, when a magnetic field is applied in-plane to the symmetry axis, the ISC transition can produce a variable PL response when the direction of the magnetic field is changed along the plane.

**Figure 6:**
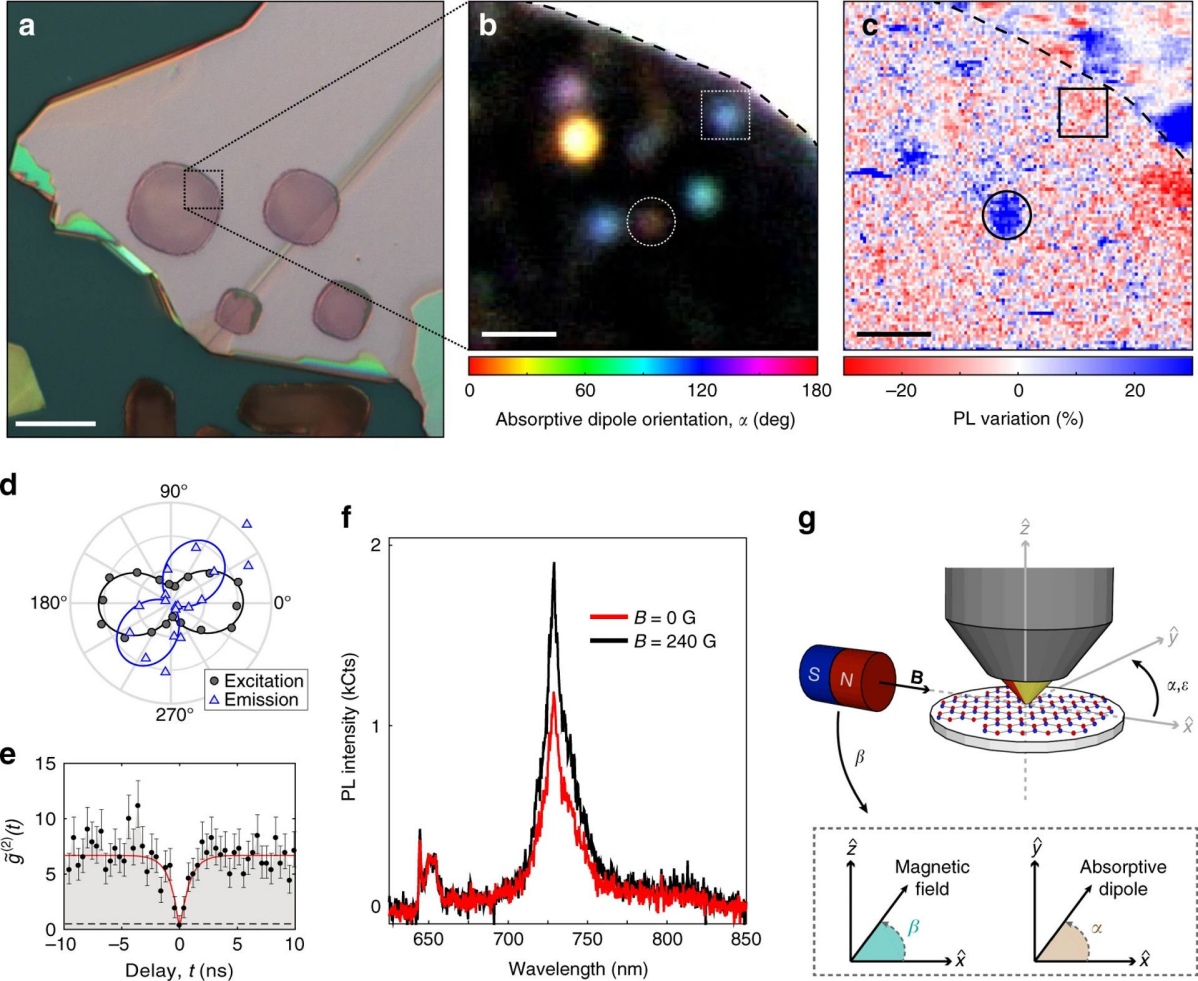
Magnetic field dependent fluorescence of an SPE in h-BN. (a) Optical microscope image of an exfoliated h-BN flake on a patterned substrate. Scale bar 10 μm. (b) PL image in absence of an applied magnetic field *B*, obtained using polarization control of the absorptive dipole orientation of suspended h-BN SPEs (represented by the white dashed box). Scale bar 1 μm. (c) PL variation image of the SPEs from area b when an in-plane magnetic field, *B* = 240 G, is applied. Blue (red) denotes higher (lower) PL when a B ≠ 0 is applied. Scale bar 1 μm. (d)–(f) Details of the SPEs circled in (b) and (c): (d) PL excitation (circles) and emission (triangles) polarization dependences with fits of the data. (e) Background corrected *g*^(2)^ function with a fit to a three-level system. (f) PL spectra with *B* = 240 G and without *B* parallel to the SPE’s absorptive dipole. (g) Illustration of the coordinate system used with β, defined as the angle of the magnetic field for the *x*–*z* plane, and α(ε), in the *x*–*y* plane, denotes the absorptive (emissive) dipole angle. Reprinted (adapted) with permission from [149], an article licensed under a Creative Commons Attribution 4.0 International License. https://creativecommons.org/licenses/by/4.0/.

In [149] (Figure 6) a *C*_2_*_v_* symmetry defect Hamiltonian (as for V_N_C_B_ or V_N_N_B_) is considered for an electronic configuration with total spin *S* where the hyperfine coupling with nuclear spins are neglected, as described by Equation 25 above. The spin–orbit coupling in h-BN is relatively weak, *g* ≈ 2, and *D* and *E* are the nonzero empirical parameters in *C*_2_*_v_*. It is to be noted that in higher-symmetry cases, such as *C*_3_*_v_* or *D*_3_*_h_*, *E* vanishes. Here the magnetic field dependence of the ISC was observed on SPEs from the photon bunching and PL emission in h-BN 400 nm thick exfoliated flakes. This is the first indication that optically addressable spin defects are present in h-BN [149]. However, the assignment to a specific defect is not possible due to the variability of the ZPL in SPEs.

In [46] the first ODMR of an SPE in single-crystal h-BN is reported with an inferred isotropic *g* ≈ 2 and a not directly observed upper bound of the ZFS ≤ 4 MHz and a hyperfine coupling at 10 MHz. The paramagnetic emitters appear to be of different origin from the SPEs previously studied and the ODMR can only be observed by 633 nm excitation and at cryogenic temperature. The spin–lattice relaxation time is of only 17 µs. The narrow and inhomogeneously broadened ODMR lines differ significantly from the single vacancy defect lines known from EPR, suggesting that the defect structure may not be due to vacancy defects but can be related to chemical addition of impurity atoms possibly introduced during exfoliation or thermal annealing. The ODMR seems more aligned with DFT predictions of point defects such as the V_N_C_B_ defect.

Gottscholl et al. [150] reported EPR and PL of the tentatively assigned negatively charged boron monovacancy, V_B_, and determined the parameters of its spin Hamiltonian. This assignment has been confirmed by ab-initio simulations [151]. The defect has D_3h_ point-group symmetry and exhibits a strong room temperature PL emission at 850 nm, observed using 532 nm laser excitation. The defect has a triplet (*S* = 1) ground state with a zero-field splitting of ≈3.5 GHz and exhibits ODMR at room temperature with a contrast of 0.75%. The Hamiltonian has *S* = 1, *g* ≈ 2, |D|/h = 3.48 GHz and a small off-axial component of the ZFS *E*/*h* = 50 MHz. The spin polarization of this center under optical pumping is also demonstrated, which is a prerequisite for coherent spin-manipulation schemes. These studies were performed in single crystal and multilayered h-BN flakes. The general conclusion is that this defect is intrinsic in nature rather than involving other external impurities, and it is very different from other observed ODMRs in h-BN [46].

### Photonics, plasmonic, optomechanics applications

A key challenge for the practical realization of an SPS is the efficient extraction of light from the quantum emitter. As discussed before, the photon collection/extraction rate from a quantum source is the product of the Purcell enhancement factor (*F*_p_) and CE. A variety of approaches for SER or Purcell enhancement as well as for efficient light collection have been employed in the last two decades. All these schemes are based on the principle of tuning the local electromagnetic environment seen by the emitted photon. One approach is to couple the emission to the strongly localized high-*Q* mode of an optical cavity [152–153]. Here, *F*_p_ ∝ (Q/V) with *V* being the mode volume. A large Purcell enhancement over the narrow spectral range can therefore be achieved using this approach. The coupling of the h-BN ensemble of quantum emitters has been demonstrated using microdisk resonators [121]. Here a hybrid h-BN and Si_3_N_4_ microdisk structure on a silicon substrate was realized by pick and place of the h-BN flakes. The microdisk had *Q* factors from 1000 to 3500. The low density of SPEs was activated by the local strain generated in the h-BN film at the resonator circumference, with emission within the whispering gallery mode volume of the microdisk. The reduced bandwidth of the ZPL of the emitters in resonance with the resonators mode was observed as well a reduction of the lifetime for tuned emitters compared to a detuned one. A moderate cavity-mediated out-coupling and Purcell enhancement (1.3) of the emission from h-BN color centers through the microdisk cavity modes could be inferred.

**Figure 7:**
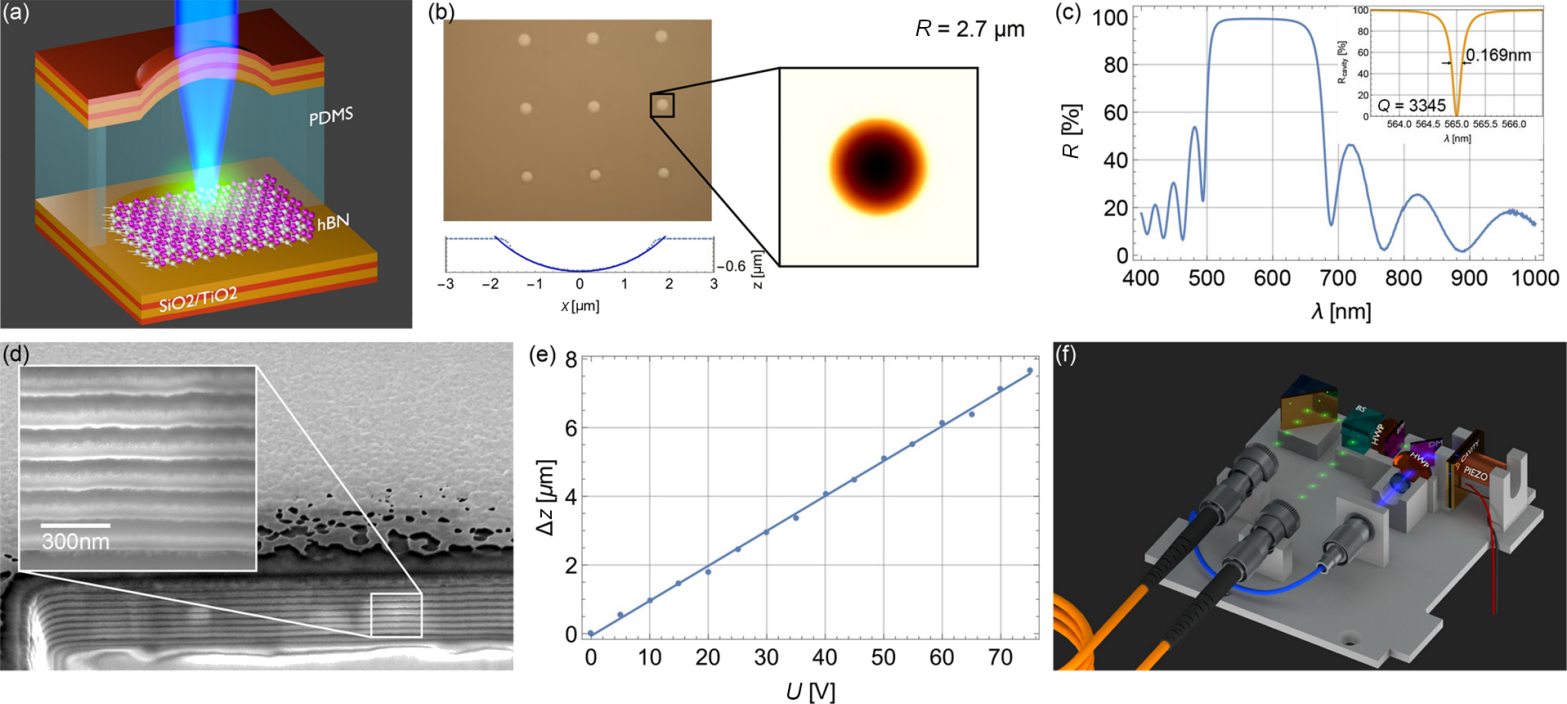
Design and fabrication of a microcavity incorporating h-BN consisting of a hemispherical and flat mirror (a). The SPE in the h-BN is aligned to emit along the confocal direction defined by the excitation laser. The cavity length is set by a PDMS spacer which is etched in the middle. (b) Optical microscope image of the array of fabricated hemispheres. The right inset shows the surface profile of the hemisphere, the bottom inset shows the height profile of the hemisphere viewed with a cross-section of 2.7 μm radius. (c) Measured reflectivity of the mirror coating of 99.2% at a wavelength of 565 nm, while the inset shows the calculated cavity reflectivity based on the used coating. (d) SEM image of the mirror stacks. The inset areas in the cross-section have a 125000× magnification. (e) The PDMS film thickness changes with driving voltage permitting a linear cavity tuning of 102 nm·V^−1^. (f) All components are shown in the scale design, including the highly focused optical excitation of the microcavity provided by a polarization-maintaining fiber (blue) coupled to the platform below the laser diode, the dichroic mirror, and a band pass filter to select the SPE, then split by a 50:50 beam splitter (BS) whose outputs are collected by multimode fibers (orange). Reprinted (adapted) with permission from [154], copyright (2019) American Chemical Society.

Vogl et al. [154] (Figure 7) shows the integration of a h-BN single quantum emitter into a tunable optical microcavity, consisting of a hemispherical and flat mirror, with a small mode volume of the order of λ^3^, a *Q* = 3345 and spectral linewidth of 0.224 nm. Multilayer h-BN flakes were exfoliated from bulk and transferred onto the cavity by using PMMA and using oxygen plasma etching to create the quantum emitters and etch the PMMA. Purcell enhancement – estimated to be around 4 – of the fluorescence was observed due to excited state lifetime reduction, with a reduction of the lifetime by a factor 2.3 and a reduction of the spectral emission from an uncoupled emitter bandwidth from 5.76 nm to the cavity linewidth. The cavity significantly narrows the spectrum, improves the SP purity, and the SP brightness reaches a count rate above 4 Mcts/s due to the improved CE of less than 2 Mcts/s of the uncoupled emitter.

Photonic crystal cavities (one-dimensional and two-dimensional photonic crystal structures) were created on suspended layers of h-BN flakes by reactive ion etching (RIE) with electron-beam-induced etching (EBIE), the latter of which is also used as direct writing for tuning the cavity in resonance to the SPE. The flakes were obtained by mechanical exfoliation. This work uses a monolithic system approach in which the photonic resonator hosts the quantum emitter, which is the best pathway to achieve strong coupling. For the two-dimensional cavity, the *Q*-factors were only 160 due to the low refractive index of the h-BN, while up to 2000 for the nanobeam cavity were achieved. SPSs were created randomly in the cavity using annealing after the fabrication. However, no coupling was observed regarding the tuning of the cavity. Medium to low *Q* factors in the range of a few 1000s have been demonstrated for h-BN-based photonic crystal cavities in both works, even if some small differences in the fabrication procedures were used [152–153]. This approach, however, has its limitations as the Purcell enhancement is limited to a very narrow spectral range. The cavity coupling is very challenging without a good control on tuning the SPEs.

Another widely used approach is to couple the emission to plasmonic/metallic resonator based structures [155]. The localized surface plasmon modes in the vicinity of the metallic resonator structures result in large field confinement over a broad emission range. This effect can permit us to achieve a very large Purcell enhancement for dipole emission coupled to these resonators over a large spectral bandwidth. The coupling of h-BN quantum emitters to plasmonic nanocavity arrays made of gold and silver nanoparticles was achieved, deterministically transferring a pre-characterized quantum emitter in h-BN onto plasmonic arrays. This resulted in a factor of two reduction in the lifetime and saturation count rates of single emitters compared to the same emitters uncoupled [156]. In [157] a nano-assembly of gold nanospheres is shown with ultrabright narrow-band quantum emitters in h-BN using an atomic force microscope (AFM) tip to precisely position the gold nanospheres in proximity to the quantum emitters. The plasmonic resonance permits observation of a PL enhancement and lifetime reduction, with a radiative QE of up to 40% and a saturated count rate above 5 Mcts/s.

Tracking the variation in the LDOS with the varying separation of the emitter from a metallic nanosphere attached to the tip of an AFM cantilever, the direct measurement of the h-BN emitter’s QE has been reported with a record of 87% for SPEs with ZPLs at 580 nm [158]. The nanoplasmonic approach has its limitations as the collection efficiencies for the plasmonic schemes are generally low due to the significantly large absorption losses in metals.

Recently, a new class of hyperbolic metamaterial structures (HMM) having an extremely anisotropic permittivity profile with metallic properties along one direction and dielectric properties along the other directions have emerged as a very promising candidate for providing an emitter with large Purcell enhancement over a very broad spectral range [159–160]. The anisotropy in the permittivity provides the structure with a hyperbolic dispersion profile with asymptotically directed large momentum, that is, high-*k* modes. These high-*k* modes have unbounded momentum values along specific directions [161]. These structures provide an emitter with a large LDOS together with high emission directionality [162–163]. Recently, Imran et al. [164] has proposed a computational design for a graphene-h-BN planar HMM structure for effective extraction of SPE from h-BN-based quantum emitters. Here the hyperbolic phonon polaritons and the natural hyperbolic properties of h-BN could be combined with the tunable behavior of graphene in the HMM structure.

In the field of integrated quantum photonic applications, various collection schemes to couple the h-BN emitter’s emission to an optical fiber with 10% coupling efficiency [165] and AlN photonic waveguides with 1.35% coupling efficiency [166] have been successfully demonstrated. In these collection schemes, the emission is channeled/tuned along with the modes of the optical waveguides without significantly altering the LDOS, which is important to build integrated quantum photonics circuits.

**Figure 8:**
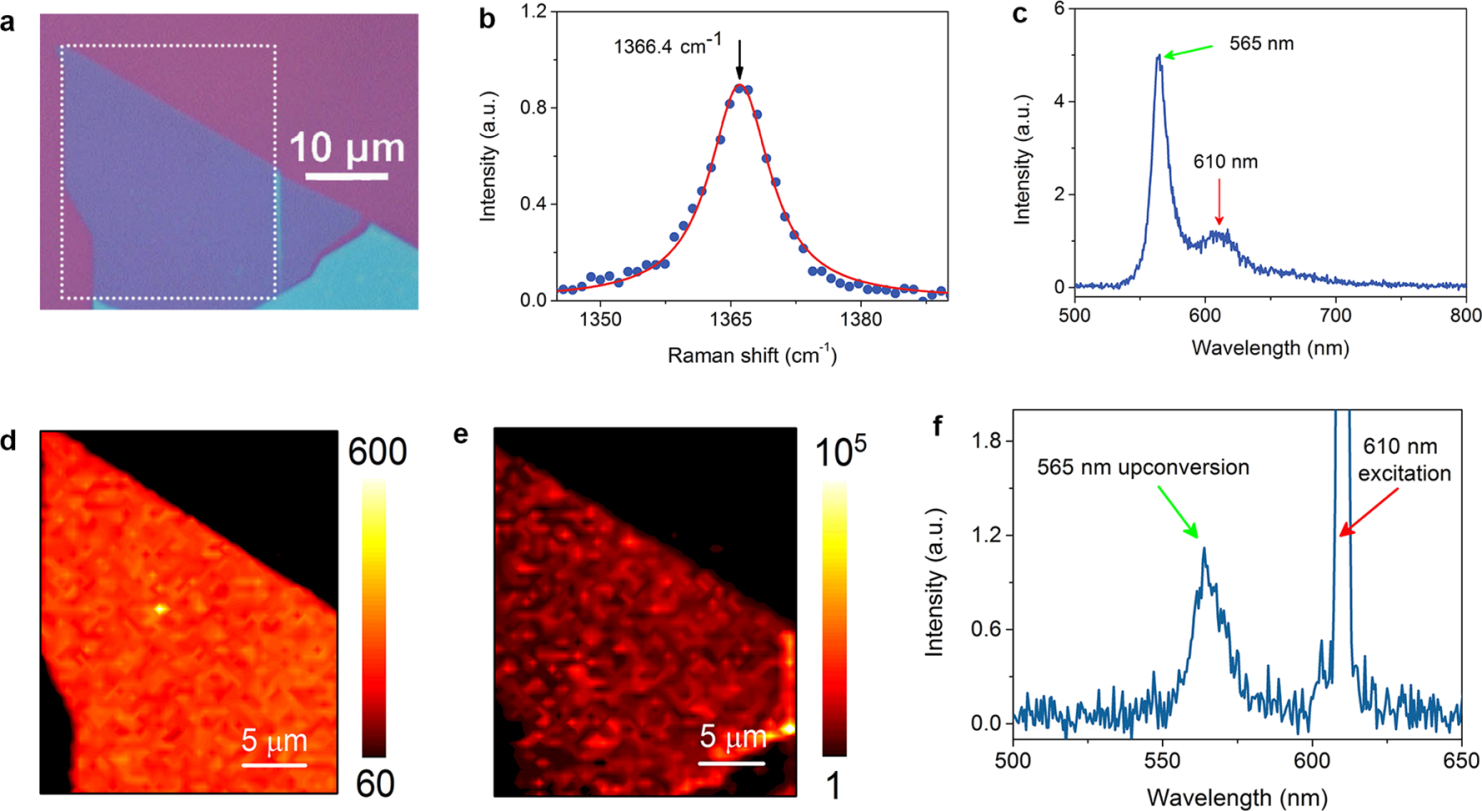
Upconversion of defects in h-BN. (a) Optical microscope image of an exfoliated h-BN flake on SiO_2_. (b) Room temperature Raman spectrum (with a 532 nm excitation wavelength) of the squared area of the h-BN flake in (a). (c) Room temperature PL spectrum of emitters in h-BN with ZPL at 565 nm and PSB at 610 nm excited at 532 nm. (d,e) Linear scale Raman mapping at 1366.4 cm^−1^ and PL mapping of the ZPL in the square in (a). (f) PL spectrum of the up-converted defects in h-BN when a 610 nm laser excites defects in h-BN, resulting in emission at 565 nm. To filter out the 610 nm excitation laser, a 600 nm short-pass filter is used. Reprinted (adapted) with permission from [167], copyright (2018) American Chemical Society.

Among the many photonic applications of h-BN defects, recently these emitters have also shown the possibility of PL frequency upconversion (an anti-Stokes process) with a significant energy gain of about 162 meV (Figure 8) [167]. This is relevant for nonlinear photonics applications in the material. The frequency upconversion process is attributed to the absorption of an optical phonon and is dependent on the excitation power, excitation wavelength, and the operating temperature.

Another interesting application is cavity optomechanics recently demonstrated using h-BN [168]. Here nanomechanical resonators consisting of h-BN beams were positioned on high mechanical *Q* silicon microdisk cavities. The thermally driven motion of the h-BN mechanical resonator between 1 and 23 MHz is read out via its interaction with a Si microdisk with a 0.16 pm/Hz sensitivity.

### Super-resolution imaging

As described in many other papers, h-BN emitters have been applied in various imaging modalities in addition to one-photon scanning confocal microscopy to localize SPEs. In addition to the one-photon excitation pathway used to characterize the SPEs, it has been shown in [169] that h-BN SPEs can be excited by two-photon absorption using a femtosecond pulsed laser at 780 nm. Two-photon scanning confocal microscopy is relevant in biophotonics as it enables imaging of bio-labels with a reduced background under excitation and detection in the biological spectral window. Further, other modalities involving wide-field microscopy and other nonlinear absorption microscopy techniques were used to better localize the emitters in the material beyond the diffraction limit.

**Figure 9:**
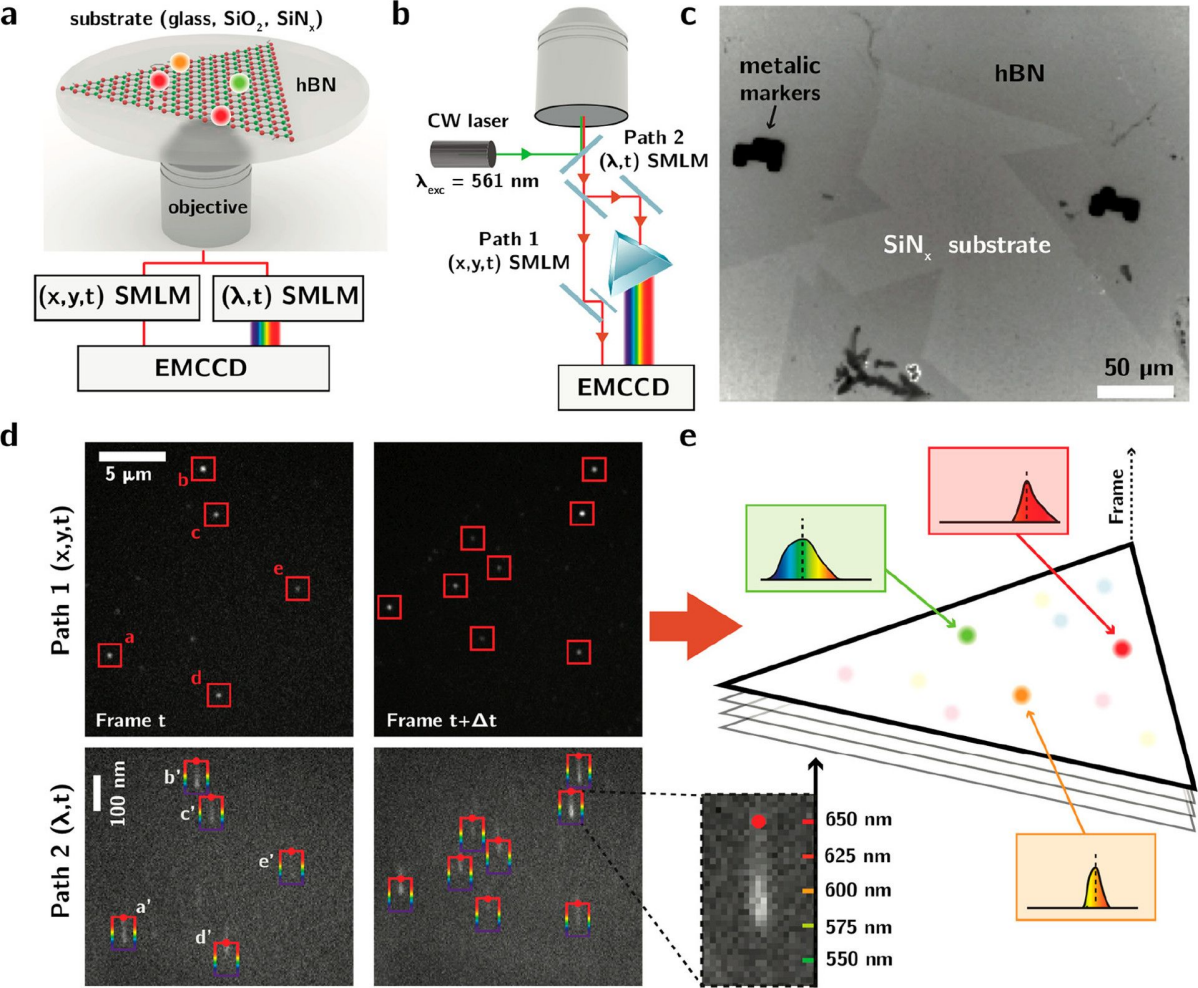
Spectral wide-field characterization of quantum emitters in h-BN. (a,b) Experimental setup schematic to provide spatial and spectral SMLM images of CVD and flakes of h-BN deposited on various substrates (glass, SiO_2_, and SiN*_x_* chips). Laser excitation is 561 nm, the PL from SPEs is collected by a high-NA objective and divided into two distinct paths (path 1,2). From spatial path 1 the diffraction-limited spot for SPEs can be localized with subpixel accuracy. Spectral path 2 has a dispersive prism to shift the PL of the SPEs based on their emission wavelength. Images from paths 1, 2 are then read on the same chip of an electron-multiplying charge-coupled camera (EMCCD) shown in (d). (c) Optical image of CVD-grown h-BN flakes after transfer onto Si/SiN*_x_* substrate. (d) Wide-field images (path 1) and spectral image (path 2) of SPEs recorded simultaneously at two subsequent time frames, *t*, and *t* + Δ*t*. The red boxes in path 1 indicate the spatial position of SPEs, while the multicolored boxes in path 2 show the corresponding images of the same SPEs after vertical dispersion by the prism. The red dots in the spectral channel corresponds to the calibrated spectral position of 650 nm for each emitter in the spatial channel (zoomed-in section of d). Images are averaged over five frames and each frame is taken with 20 ms exposure. (e) Reconstruction of a super-resolution spectral image is obtained by summing up successive frames to provide the sub-diffraction spatial positions of emitters with their spectral information. Reprinted (adapted) with permission from [174], copyright (2019) American Chemical Society.

Stochastic localization microscopy or single-molecule localization microscopy (SMLM), has been applied to monolayers of CVD-grown h-BN transferred to the SiN*_x_* membrane as described in [170]. The fluorescence emission of diffraction-limited defects in the material with ≈11 nm localization imaging was distinguished. This is achieved by inducing an “on” and “off” emission of quantum emitters subject to a specific excitation wavelength, either due to a photo-ionization mechanism or an electron tunneling process, as previously shown in diamond and nanodiamond NV centers [171–173]. While STM has already been used to directly visualize single defects and their electronic structures in addition to aberration-corrected TEM to assign created defects in h-BN [65], both methods require special sample preparations are limited to a very small field of view. This can induce other defects due to the high energy that can accelerate electrons and thus may not be compatible with some applications in both quantum information processing and bioimaging, where optical methods that can directly image individual defects are preferred. Here the photo-ionization achieved with 561 nm excitation is attributed to switching of the V_B_ neutral state to its negative charge state. This first emission is the dominant broadband emission in the 600 nm that is less bright and attributed to as-grown defects or those induced during electrochemical transfer, while the second emission is red-shifted towards 615 nm and attributed to activated single defects of the negative V_B_. In this sample, V_B_ was dominant over V_N_ based on the TEM images. Here also larger extended defects in the material can include emission from V_N_V_B_ [65] or V_B3N1_ [127]. In [174] SMLM was used to study the difference of the ensemble and single quantum emitters in CVD and bulk exfoliated flakes of h-BN. This is based on photo-switching between "on" and "off" states with added spectral selectivity as described in Figure 9. Two families of emitters were found: one with emission spectra centered approximately around ≈585 nm (“green emitters”) for both CVD and flakes, and one at ≈640 nm (“red emitters”) for the flakes and in the region 610−650 nm for CVD, resulting in wider emission due to defects introduced during substrate transfer. For more deterministically induced defects in the flakes, they were exposed to 30 s of oxygen plasma treatment, and the green emitters were mostly generated. All these emitters were blinking and also undergoing photo-bleaching when created using oxygen plasma. The study also provides strategies to achieve a reduction of blinking and increased photostability via the use of encapsulation of defects or reduction of charge traps using thermal annealing. Regardless of the similarity with other studies in terms of the emitter spectral classification, no clear attribution was possible from this study.

h-BN nanoparticles produced by a cryogenic exfoliation technique with diameters of the 3 nm are biocompatible and embed SPEs with similar PL as in h-BN flakes. These materials have been used for super-resolution imaging by SMLM [175] showing their potential application as biomarkers.

In h-BN flakes, the ZPLs with SPEs are grouped around 560 nm, 580 nm, 640 nm, and 714 nm. Different point-like defects could be responsible for each group with the crystal lattice locally strained or changed otherwise, thus explaining the spread around these wavelengths. However, current methods to characterize flakes in terms of these groups cannot distinguish between few and multilayers as the direct imaging at the atomic scale using STEM is limited to a few layers, thus retaining information from all layers. In [129] SPEs were studied layer-by-layer by controlled etching. Here mechanically exfoliated flakes for h-BN were produced, then transferred to a Si substrate terminated with a layer of thermally grown SiO_2_, where they were subjected to oxygen plasma treatment and subsequent annealing at 850 °C in Ar to create defects. The emitters created with these methods are more likely to form at flake edges and grain boundaries, and longer ZPLs (714 nm) were not formed. Layer-by-layer etching of h-BN is also achieved using oxygen plasma treatment, and monolayers to 11 layer flakes were controllably formed. After the initial confocal characterization of SPEs in multilayers flakes, a total of 93 SPEs were imaged and subsequent layer-by-layer etching was performed. The emitters were rechecked in the confocal microscope to determine whether they survived or not and monitored until they disappeared to determine the locations where the emitters were created. It was determined that the SPEs were generated close to the surface and well-localized within one layer with no appreciable inter-layer interaction due to the abrupt PL disappearance rather than attenuation. The SPEs generated by oxygen plasma had a maximum probability of being in layer *N* = 3.8 on average. As the process could generate new emitters during etching, this situation was not counted in the survey. The observation, however, is valid for the specific methods of defect creation and does not necessarily apply to other physical fabrication methods. For example, when defects were generated by high energy electron irradiation they were located throughout the entire crystal thickness. While the defects created using oxygen plasma can be associated with oxygen vacancy complexes, the ones generated using electron irradiation are damage defects such as V_B_ or V_N_.

Other deterministic super-resolution methods such as reversible saturable optical fluorescence transitions and ground state depletion microscopy were applied to specific long ZPL (780 nm) quantum emitters, reaching the best subdiffraction resolution of 87 nm (one excitation beam) and 63 nm (using two excitation beams) [176]. The two methods were based on forcing the emitters to become dark using either stimulated emission excitation at a longer wavelength or by high power one beam excitation, forcing the emitters to a long-lived metastable state.

## Conclusion

SPEs in h-BN have been abundantly studied in various types of materials, namely 0D, 1D, 2D, and 3D and are based on different material fabrication methods (from exfoliation to CVD). The emitters show a large variety of ZPLs and photo-physical properties, albeit with some similarity that permits grouping of their emission and possible association to common points defects. These are primarily the monovacancy (V_B_ and V_N_), complex vacancy (V_B3N1_), antisite (V_N_N_B_), substitutional carbon antisite (V_N_C_B_), divacancy (V_B_V_N_), or other oxygen and carbon substitutionals. As the SPEs are strongly polarized in excitation and absorption for the in-plane electric field, the symmetry of the point defects involved has to be low. Ab-initio simulations of common defects in h-BN to match the experimental results have been performed and ZPLs tend to be reproduced. However, several contradictions emerge with experimental investigation. Various physical and chemical fabrication methods (based on electrons, ions, neutrons, laser irradiation, and chemical etching and annealing) appear to be useful for their controlled formation up to quasi-deterministic methods. These latter are based on strain control and focused ion beam techniques, which produces different point defects from those most likely attributed to some sort of vacancy related defects due to physical fabrication methods.

In general, the emission is very bright, up to 7000 kcts/s at saturation, in multilayer flakes. Using specific treatments, the SPE purity has reached values of <0.1 and with a linewidth of 55 MHz, which corresponds to a Fourier transform limited SPS up to room temperature. Both electrical, strain and acoustic wave control appear to be excellent in the flakes. Fewer experiments have been performed on a more controlled initial material with higher purity obtained from CVD h-BN. In this space, preliminary works seem to indicate that material fabrication is a key element to improve SPE spectral control and purity. However, methods to transfer the CVD h-BN to other substrates without introducing defects are under study.

Particular advantages of SPE in h-BN include emission over a broad spectral region from UV–vis–NIR, room temperature operation and robustness for high-temperature operation, high brightness and high QE in some cases, controllability of the excitation and emission polarization, tunable emission, and coherent control of the optical transition using on-resonance excitation. Additionally, optically detected magnetic resonance and electron spin coherence were observed in an ensemble of emitters attributed to the V_B_, which is one of the defects likely formed using neutron irradiation. There are now approaches indicating their possible controllable engineering, although the lack of identification of each individual group of defects makes on-demand engineering of specific emitters for integration in the photonics cavity difficult. Nevertheless, the large variety of nanomaterials showing SPEs provides an interesting platform for nanophotonics and nanoplasmonics as well as applications in super-resolution imaging and bioimaging markers. These last applications are the most appealing due to the variety of nanomaterials based on h-BN available with SPEs as well as their hybrid integration in quantum devices (waveguides, integrated photonics circuits, photonics, and nanoplasmonic cavities, and optomechanics) that could reveal a viable approach for quantum technology.
